# High-coverage whole-genome sequencing of the expanded 1000 Genomes Project cohort including 602 trios

**DOI:** 10.1016/j.cell.2022.08.004

**Published:** 2022-09-01

**Authors:** Marta Byrska-Bishop, Uday S. Evani, Xuefang Zhao, Anna O. Basile, Haley J. Abel, Allison A. Regier, André Corvelo, Wayne E. Clarke, Rajeeva Musunuri, Kshithija Nagulapalli, Susan Fairley, Alexi Runnels, Lara Winterkorn, Ernesto Lowy, Human Genome Structural Variation Consortium, Evan E. Eichler, Jan O. Korbel, Charles Lee, Tobias Marschall, Scott E. Devine, William T. Harvey, Weichen Zhou, Ryan E. Mills, Tobias Rausch, Sushant Kumar, Can Alkan, Fereydoun Hormozdiari, Zechen Chong, Yu Chen, Xiaofei Yang, Jiadong Lin, Mark B. Gerstein, Ye Kai, Qihui Zhu, Feyza Yilmaz, Chunlin Xiao, Paul Flicek, Soren Germer, Harrison Brand, Ira M. Hall, Michael E. Talkowski, Giuseppe Narzisi, Michael C. Zody

**Affiliations:** 1 New York Genome Center, New York, NY 10013, USA; 2 Program in Medical and Population Genetics, Broad Institute of MIT and Harvard, Cambridge, MA 02142, USA; 3 Center for Genomic Medicine, Massachusetts General Hospital, Boston, MA 02114, USA; 4 Department of Neurology, Massachusetts General Hospital and Harvard Medical School, Boston, MA 02114, USA; 5 McDonnell Genome Institute, Washington University School of Medicine, St. Louis, MO 63108, USA; 6 Department of Medicine, Washington University School of Medicine, St. Louis, MO 63110, USA; 7 Outlier Informatics Inc., Saskatoon, SK S7H 1L4, Canada; 8 European Molecular Biology Laboratory, European Bioinformatics Institute, Wellcome Genome Campus, Hinxton, Cambridge CB10 1SD, UK; 9 Stanley Center for Psychiatric Research, Broad Institute of MIT and Harvard, Cambridge, MA 02142, USA; 10 Center for Genomic Health, Yale University School of Medicine, New Haven, CT 06510, USA; 11 Department of Genetics, Yale University School of Medicine, New Haven, CT 06520, USA

**Keywords:** 1000 Genomes Project, whole-genome sequencing, population genetics, SNV, INDEL, structural variation, reference imputation panel, trio sequencing

## Abstract

The 1000 Genomes Project (1kGP) is the largest fully open resource of whole-genome sequencing (WGS) data consented for public distribution without access or use restrictions. The final, phase 3 release of the 1kGP included 2,504 unrelated samples from 26 populations and was based primarily on low-coverage WGS. Here, we present a high-coverage 3,202-sample WGS 1kGP resource, which now includes 602 complete trios, sequenced to a depth of 30X using Illumina. We performed single-nucleotide variant (SNV) and short insertion and deletion (INDEL) discovery and generated a comprehensive set of structural variants (SVs) by integrating multiple analytic methods through a machine learning model. We show gains in sensitivity and precision of variant calls compared to phase 3, especially among rare SNVs as well as INDELs and SVs spanning frequency spectrum. We also generated an improved reference imputation panel, making variants discovered here accessible for association studies.

## Highlights

• Expansion of the 1000 Genomes Project (1kGP) resource to include 602 trios
• High-coverage whole-genome sequencing of the expanded 1kGP cohort
• Discovery of more rare SNVs as well as INDELs and SVs across the frequency spectrum
• Generation of an improved and accessible reference imputation panel

## Graphical abstract

**Figure:**
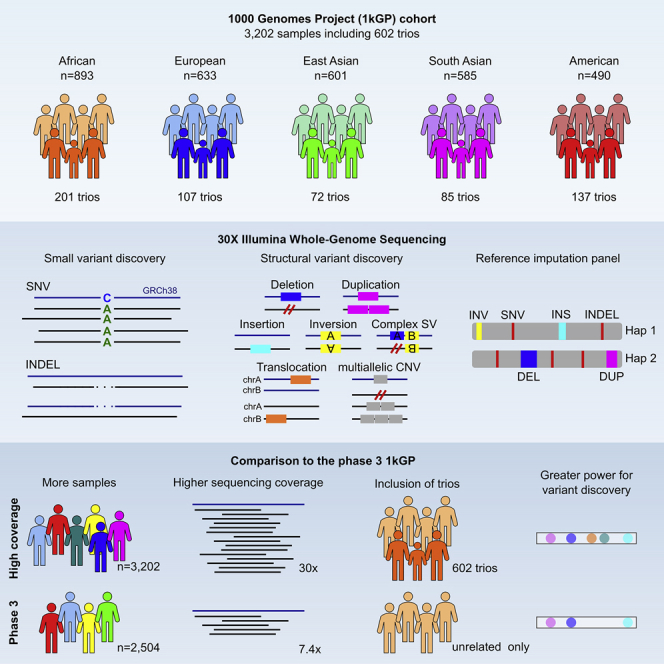

## Teaser

High-coverage whole-genome sequencing (WGS) of the expanded 1000 Genomes Project (1kGP) cohort including 602 trios led to the discovery of additional rare non-coding single-nucleotide variants (SNVs), as well as coding and non-coding short insertions and deletions (INDELs) and structural variants (SVs) spanning the allele frequency spectrum compared to the original 1kGP resource based primarily on low-coverage WGS.

## Introduction

The 1000 Genomes Project (1kGP) was the first large-scale whole-genome sequencing (WGS) effort to deliver a catalog of human genetic variation (Sudmant et al., 2015; The 1000 Genomes Project Consortium, 2010, 2012, 2015). The project sampled participants from 26 populations across five continental regions of the world. It culminated in 2015 with publication of the final, phase 3 variant call set (Sudmant et al., 2015; The 1000 Genomes Project Consortium, 2015) consisting of 2,504 unrelated samples, a subset of which is from the HapMap collection (The International HapMap 3 Consortium, 2010). The phase 3 call set was generated based on the combination of low-coverage WGS (mean depth 7.4X), high-coverage whole-exome sequencing (WES, mean depth 65.7X), and microarray genotyping data from lymphoblastoid cell line (LCL) samples. It included 84.7 million single-nucleotide variants (SNVs), and 3.6 million short insertions and deletions (INDELs), as well as a separate set of 68,818 structural variants (SVs; alterations ≥50 bp). The 1kGP resources have been collectively cited over 18,000 times to date and have been utilized for foundational applications such as genotype (GT) imputation, expression quantitative trait loci (eQTL) mapping, variant pathogenicity prioritization, population history, and evolutionary genetics studies (Almeida et al., 2014, Hara et al., 2014, Horikoshi et al., 2015, Huang et al., 2015, Khurana et al., 2013, Kircher et al., 2014, Lappalainen et al., 2013, Ritchie et al., 2014, The CARDIoGRAMplusC4D Consortium et al., 2015, Zheng-Bradley and Flicek, 2017). While the phase 3 dataset captured the vast majority of common SNVs (minor-allele frequency [MAF] >1%) in the population (>99%) (The 1000 Genomes Project Consortium, 2015), detection of rare SNVs (MAF ≤1%) as well as INDELs and SVs across the entire frequency spectrum was limited due to low sequencing coverage outside of the coding genome as well as shortcomings in INDEL and SV discovery algorithms that existed at the time of phase 3 data analysis.

Here, we present high-coverage WGS and comprehensive analyses of the original 2,504 1kGP samples, as well as of 698 additional related samples that now complete 602 trios in the 1kGP cohort. We sequenced LCL-derived DNA from the expanded cohort of 3,202 samples to a targeted depth of 30X genome coverage using Illumina NovaSeq 6000 instruments. We aligned reads to the GRCh38 reference and performed SNV/INDEL calling using GATK HaplotypeCaller (Poplin et al., 2017). We also discovered and genotyped a comprehensive set of SVs, including insertions (INSs), deletions (DELs), duplications (DUPs), inversions (INVs), and multiallelic copy number variants (mCNVs), by integrating multiple algorithms and analytic pipelines, including GATK-SV (Collins et al., 2020), svtools (Larson et al., 2019), and Absinthe (Corvelo et al., 2021). Comparison with previous low-coverage sequencing performed in phase 3 of the 1kGP demonstrates significant improvements in sensitivity and precision of variant calls, highlighting that the resequencing effort and expansion of the cohort to include trios brought significant value to the resource.

One of the major applications of the phase 3 1kGP call set has been its widespread use as a reference panel for variant imputation in sparse, array-based genotyping data with a goal of improving the statistical power of downstream genome-wide association studies (GWAS) and facilitating fine-mapping of causal variants. As part of this publication, we release an improved reference imputation panel based on the high-coverage WGS consisting of SNV, INDEL, and SV calls across the 3,202 1kGP samples, including full trios.

Since the completion of phase 3, much larger WGS datasets have been released such as the Genome Aggregation Database (gnomAD; 76,156 WGS samples) (Karczewski et al., 2020), Trans-Omics for Precision Medicine (TOPMed, ∼180,000 samples) (Taliun et al., 2021), All of Us (∼100,000 samples), or the UK Biobank (UKBB, 200,000 samples) (Halldorsson et al., 2022). Unlike the 1kGP, these resources have restrictions on public data sharing as they are often linked to clinical data, which amplifies privacy concerns. In contrast, samples within the 1kGP cohort have been consented for full public release of genetic information, which allows for unrestricted sharing of the complete sample-level GT data. This enables granting access to a downloadable reference imputation panel, as well as use of the dataset for methods development and benchmarking, among other applications. A small subset of the 602 pedigrees that are now part of the expanded 1kGP cohort have been sequenced previously as part of various efforts, such as Platinum Genomes (Eberle et al., 2017), Complete Genomics (The 1000 Genomes Project Consortium, 2015), and the Human Genome Structural Variation Consortium (HGSVC) (Chaisson et al., 2019; Ebert et al., 2021); however, we have sequenced nearly all 1kGP trios at high coverage and jointly analyzed them for the discovery and genotyping of genomic variation across the size and frequency spectrum. We make all the data generated publicly available without restriction and envision it becoming the *de facto* public resource for the worldwide genomics and genetics community.

## Results

### Small variation across the 3,202 1kGP samples

**Figure 1:**
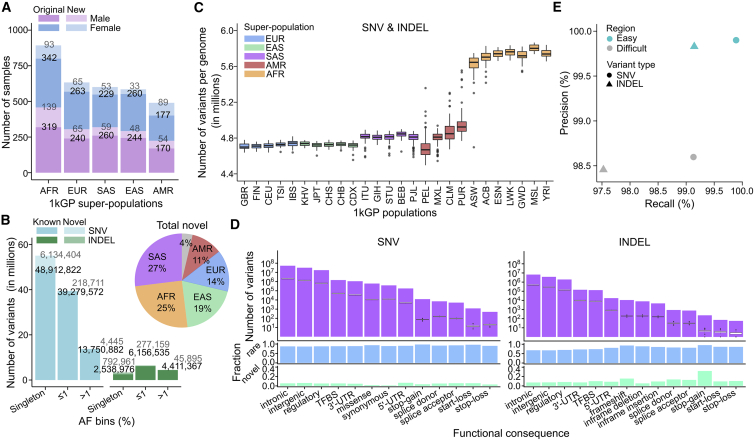
SNV/INDEL discovery in the high-coverage WGS data across the 3,202 1kGP samples (A) Counts of samples stratified by sex and super-population. Original: 2,504 original 1kGP samples. New: 698 newly added samples. (B) Cohort-level alternate allele counts of SNVs and INDELs across the 3,202 samples, stratified by AF bins. Novel/known: sites absent from/present in dbSNP build 155. AF was estimated based on the 2,504 unrelated samples. Pie chart: breakdown of all novel variants by the super-population ancestry. Gray area in the pie chart: novel sites that were called in more than one super-population. (C) Count of small variant loci per genome, stratified by population. See also Figures S1A–S1C. (D) Predicted functional SNVs and INDELs (autosomes). Top row: cohort-level counts (purple bar plot) overlaid with distributions of sample-level counts (boxplots) across the 2,504 unrelated samples. Middle row: fraction of rare (MAF ≤1%) SNVs and INDELs among the predicted functional sites. Bottom row: fraction of novel SNVs and INDELs among the predicted functional sites. See also Figures S1G and S1H. (E) Precision versus recall computed relative to the GIAB truth set v3.3.2, stratified by easy and difficult regions of the genome. See also Figure S1D. Super-population ancestry labels: EUR, European; AFR, African; EAS, East Asian; SAS, South Asian; AMR, American. Descriptions of population labels are in Table S1.

Using the Illumina NovaSeq 6000 System, we performed WGS of the original 2,504 1kGP unrelated samples and an additional 698 related samples. This completed 602 parent-child trios in the 1kGP cohort and brought the total number of sequenced and jointly genotyped samples to 3,202 (Figure 1A; Table S1). At the cohort level, we discovered a total of 117,175,809 small variant loci, which represent 125,484,020 distinct alternate alleles, including 111,048,944 SNVs and 14,435,076 INDELs (Table 1). Across all SNVs and INDELs, there are 58,379,163 (47.6%) singletons (allele count [AC] = 1), 45,931,977 (37.5%) rare (allele frequency [AF] ≤ 1%), and 18,212,589 (14.9%) common (AF > 1%) alleles, as defined using AF estimates based on unrelated samples in the cohort (Figure 1B). Out of all small variants, 7,473,575 (5.9%) represent novel alleles, defined here as not reported in SNP database (dbSNP) build 155 (Sherry et al., 1999) (Figure 1B). 92.7% of novel variants are singletons, and most are specific to a single super-population ancestry, with the highest fraction being specific to the South Asian ancestry (SAS, 27%), followed by African (AFR, 25%), and East Asian (EAS, 19%) ancestry group (Figure 1B). Although AFR genomes have more variants than SAS, recent large-scale WGS efforts to sequence more AFR genomes, such as TOPMed (∼51,000 AFR genomes compared to ∼15,000 SAS and EAS genomes combined), which now accounts for most variants in dbSNP (Taliun et al., 2021), are likely contributing to the slightly lower proportion of novel variants discovered here being specific to the AFR ancestry group. Overall, 6.6% (n = 7,676,044) of small variant loci are multiallelic across the 3,202-sample cohort. These multiallelic loci contain 6,937,157 SNV and 9,022,437 INDEL alleles of which 6.9% SNVs and 5.6% INDELs are novel.

**Table 1:** Summary of variant counts in the high-coverage 1kGP call set at the cohort and sample level

Variant type	# variants across 3,202 samples	Average # variants per sample					
	Total	All	AFR	EUR	SAS	EAS	AMR
Total small variants	125,484,020	4,952,915	5,623,313	4,645,189	4,736,023	4,651,279	4,754,817
SNV	111,048,944	4,080,992	4,653,521	3,818,951	3,896,324	3,822,328	3,911,413
IN-DEL	8,010,854	451,277	503,995	426,940	433,635	428,078	435,976
IN-INS	6,424,222	420,646	465,797	399,298	406,064	400,873	407,428
Total SVs	173,366	9,679	10,742	9,176	9,304	9,251	9,363
DEL	90,259	4,232	4,715	4,001	4,066	4,035	4,089
DUP	28,242	1,207	1,322	1,153	1,168	1,155	1,178
mCNV	673	425	433	422	419	425	419
INS	49,693	3,534	3,971	3,329	3,378	3,361	3,403
INV	920	68	71	66	67	67	66
CPX	3,568	213	230	205	206	208	208
CTX	11	0	0	0	0	0	0

Super-population ancestry labels: EUR, European; AFR, African; EAS, East Asian; SAS, South Asian; AMR, American. Small variant types: SNV, single-nucleotide variant; IN-DEL, short deletion; IN-INS, short insertion. SV types: DEL, deletion; DUP, duplication; mCNV, multiallelic copy number variant; INS, insertion; INV, inversion; CPX, complex SV; CTX, inter-chromosomal translocation.

To better characterize our variant calls, we divided the genome into easy- and difficult-to-sequence regions (see STAR Methods), as defined by the Genome in a Bottle (GIAB) Consortium (Krusche et al., 2019). The difficult regions constitute only 20% of the genome but they contain a disproportionately high fraction of all multiallelic sites (74.8% compared to 22.4% of all biallelic sites). Additionally, difficult regions are also enriched for INDEL loci, containing 64.3% of INDEL as compared to 23.1% of SNV loci. The enrichment for multiallelic and INDEL calls in difficult regions is consistent with expectation, as these regions mostly consist of low complexity and repetitive elements where alignment of sequencing reads is particularly challenging and where INDELs are known to typically form (Montgomery et al., 2013).

**Figure S1:**
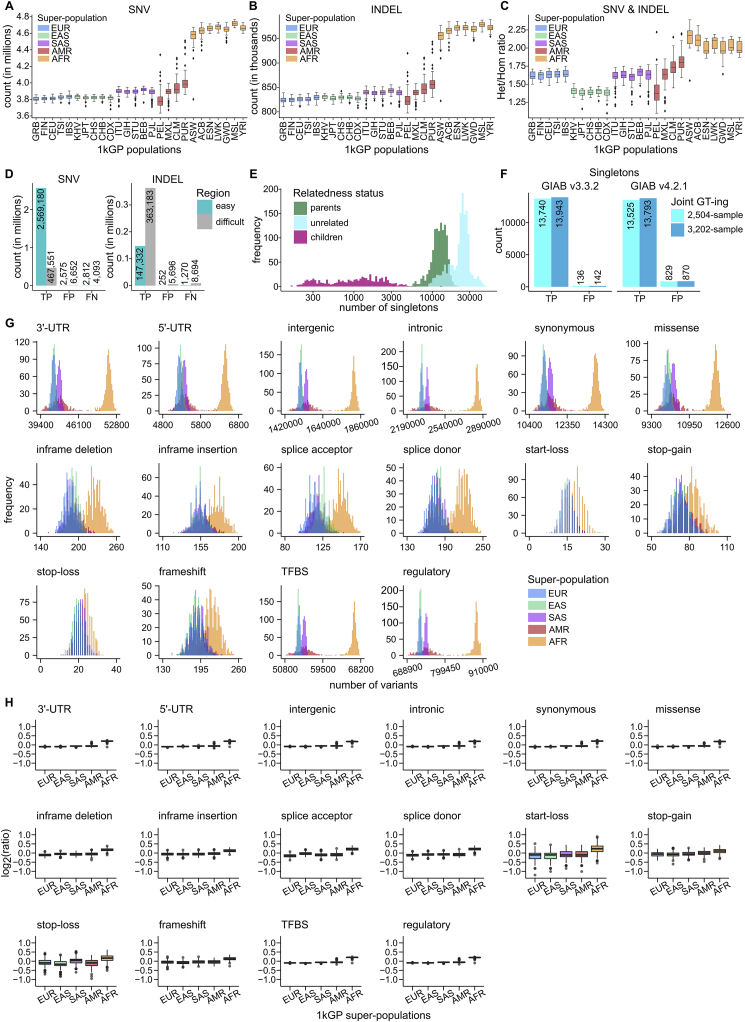
Evaluation of small variant calls, related to Figure 1 Sample-level counts of SNVs **(A)** and INDELs **(B)**, stratified by super-population. **(C)** Sample-level Het/Hom ratios across small variants, stratified by super-population. **(D)** Counts of true positive (TP), false positive (FP), and false negative (FN) SNV and INDEL calls in easy and difficult regions of the genome (GIAB v3.3.2 high confidence regions only). **(E)** Sample-level singleton (sites with AC = 1 across 3,202 samples) counts, stratified by relatedness status. **(F)** Counts of true positive (TP) and false positive (FP) singletons in NA12878 relative to either the GIAB v3.3.2 or GIAB v4.2.1 truth set (GIAB high confidence regions only). Due to the presence of NA12878’s parental samples in the expanded cohort, the analysis using the 3,202-sample 1kGP call set is based on both *de novos* and inherited variants private to the NA12878 trio. **(G)** Sample-level counts of predicted functional small variants, stratified by super-population. Reported counts are across the 2,504 unrelated samples only. **(H)** Distributions of log2(ratios) of sample-level counts from (G) normalized by the mean count across the 2,504 unrelated samples. Super-population ancestry labels: European (EUR), African (AFR), East Asian (EAS), South Asian (SAS), American (AMR). Descriptions of population labels are in Table S1. Panels E, G, H are based on autosomes

At the sample level, we called an average of 4,952,915 small variants (Table 1). As expected, the average number of variant sites was the highest in the individuals from populations with AFR ancestry, while individuals belonging to the admixed populations with American ancestry (AMR) displayed the highest variability in the number of variants (Figures 1C and S1A–S1C).

### Predicted functional consequence of small variants

To assess functional consequences of SNVs and INDELs in the high-coverage call set, we annotated them using the Ensembl Variant Effect Predictor (VEP) (McLaren et al., 2016). Across the unrelated samples, we found a total of 605,896 missense, 384,451 synonymous, and 36,520 predicted loss-of-function (pLoF) mutations, defined here as stop-gain (n = 12,181), frameshift (n = 10,850), and splice mutations (n = 13,489) (Figure 1D). Depending on the functional consequence category, 86%–97% and 67%–95% of predicted functional SNVs and INDELs, respectively, are rare (MAF ≤1%), with 1%–7% SNVs and 5%–32% INDELs being novel (i.e., absent from dbSNP build 155) (Figure 1D). At the sample level, we found on average 10,625 missense, 11,956 synonymous, 76 stop-gain, 195 frameshift, and 314 splice mutations without applying MAF filtering (Figures 1D; Table S3). At MAF ≤1%, each sample carries on average 11 stop-gain, 18 essential splice, and 14 frameshift mutations. These cohort- and sample-level counts are consistent with previous reports (Karczewski et al., 2020; Taliun et al., 2021). As expected, AFR samples carry the highest counts of variants across all functional categories as compared to other populations (Figure S1G; Table S3), with magnitudes of difference between populations being similar across high- and low-impact functional categories (Figure S1H).

### False discovery rate among small variants

We determined the false discovery rate (FDR) of the high-coverage call set by comparing GT calls in sample NA12878 to the GIAB NA12878 truth set v3.3.2 (Zook et al., 2019) in the high confidence regions of the genome. Using this approach, the estimated FDR is 0.3% for SNVs and 1.15% for INDELs. We observed ∼10-fold lower FDR (= 1 − precision) in the easy as compared to difficult subsets of the high confidence regions for both SNVs (0.10% versus 1.40%, respectively) and INDELs (0.17% versus 1.54%) (Figures 1E and S1D). In the easy regions, sensitivity of SNV and INDEL calls reached 99.89% and 99.14%, respectively, whereas in difficult regions it was 99.13% for SNVs and 97.53% for INDELs (Figures 1E and S1D).

**Figure S2:**
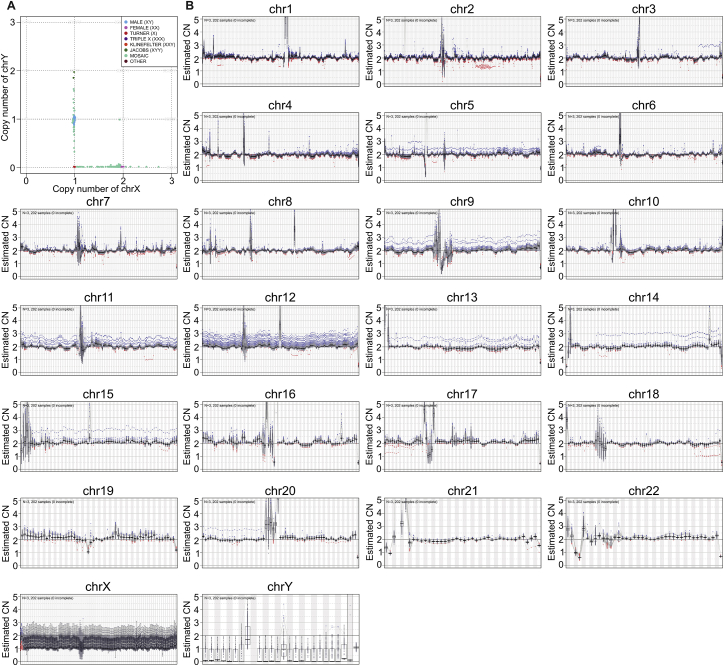
Ploidy of each chromosome across the 3,202 samples, related to Figure 1 **(A)** Ploidy of allosomes. **(B)** Copy number (CN) of each chromosome. Each dot represents a copy number of the 1Mbp bin in a sample. Blue dots are samples with copy gain and red dots represent copy loss

We separately analyzed the subset of small variants that tends to be the most enriched for false positive calls, namely the singletons (variants with AC = 1 across the entire 3,202-sample cohort). Due to the mixed nature of the expanded 1kGP cohort, which now includes both unrelated as well as related samples, the number of singletons per genome varies depending on the sample’s relatedness status, with children having the fewest singletons (mean of 1,340) followed by parents (mean of 12,365) and then unrelated samples (mean of 23,197) in the cohort (Figure S1E). Singletons among children represent putative *de novo* mutations (DNMs). The expected number of germline DNMs is ∼100 per child (Jónsson et al., 2017; Kong et al., 2012), which suggests that the mean number of singletons among children exceeds the expectation by about a factor of 10, although this varies rather widely from sample to sample (Figure S1E). Given that all 1kGP samples are derived from LCLs of various ages, these additional singletons likely represent somatic artifacts from cell-line propagation (Ng et al., 2021), as well as some false positive calls. As evidence of the presence of somatic artifacts, we observed aneuploidy of allosomes in 0.94% of the samples and sub-chromosomal level mosaic CNVs on multiple autosomes (Figure S2). This agrees with findings from the Polaris project (Illumina Inc., 2019).

For singletons in sample NA12878, the estimated FDR is 1.01% based on comparison to the GIAB truth set v3.3.2 (Zook et al., 2019). This is consistent with an independent estimation based on NA12878 from the jointly genotyped set of just the 2,504 unrelated samples where FDR is 0.98% (see STAR Methods; Figure S1F). Additionally, we evaluated singletons against the recently released GIAB truth set v4.2.1 (Wagner et al., 2022). Thanks to inclusion of additional technologies such as PacBio-HiFi and 10X Genomics, the GIAB v4.2.1 truth set excludes some of the calls believed to be mosaic variants that arose due to cell-line propagation that were present in the GIAB v3.3.2 truth set. Based on this comparison, the FDR among singletons is 5.93% (analysis based on NA12878 from the 3,202-sample joint call set) or 5.78% (analysis based on NA12878 from the 2,504-sample joint call set) (see STAR Methods; Figure S1F). This indicates that ∼5% of singleton calls in the high-coverage call set appear to be truly present in the cell lines but may not represent true population variants or even real DNMs in the original donors, highlighting potential shortcomings of using cell lines derived DNA for this study.

### Structural variation across the 3,202 1kGP samples

**Figure S3:**
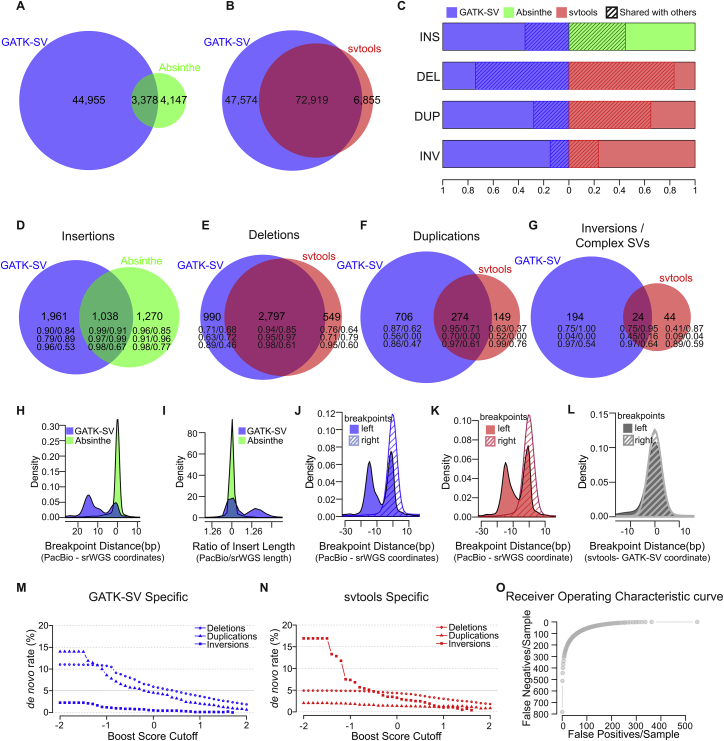
Benchmark of GATK-SV, svtools, and Absinthe, related to Figure 2 **(A)** Overlap of insertion sites between GATK-SV and Absinthe call sets. **(B)** Overlap of SV other than insertions between the GATK-SV and svtools call set. **(C)** Overlap of SV sites of each type between GATK-SV, svtools, and Absinthe. **(D)** Overlap of insertions in each genome between GATK-SV and Absinthe. **(E-G)** Overlap of deletions (E), duplications (F), inversion and complex SVs (G) in each genome between GATK-SV and svtools. The integers in (D-G) represent count of SVs per sample, followed by proportion of SVs validated by VaPoR/proportion of SVs assessable by VaPoR in the second row, proportion of SVs supported by PacBio SVs in Ebert et al., (2021)/proportion of SVs supported by PacBio SVs in Chaisson et al. (2019) in the third row, and transmission rate/rate of biparentally inherited SVs in the fourth row. **(H-I)** Precision of the insertion breakpoint (H) and length (I) assessed against PacBio assemblies. **(J-K)** Precision of the SV breakpoints in GATK-SV (J) and svtools (K) call sets assessed against PacBio assemblies. **(L)** Breakpoint distance of SVs shared by GATK-SV and svtools. **(M-N)***de novo* rate of SVs in GATK-SV (M) and svtools (N) call set when filtered at different boost score cutoffs. **(O)** False positives and false negatives in the GATK-SV and svtools call sets when filtered at different boost score cutoffs

**Figure 2:**
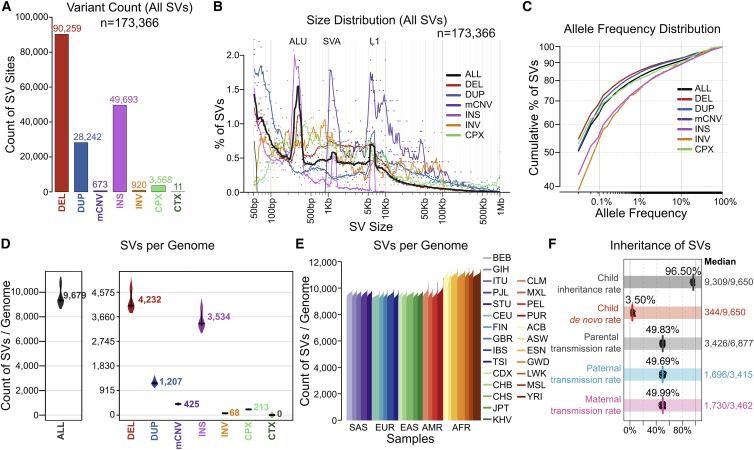
SV discovery in the high-coverage WGS data across the 3,202 1kGP samples (A–C) The count (A), size distribution (B), and allele frequency distribution (C) of each SV class. (D–F) The mean per sample count of SVs by variant class (D) and ancestral population (E) is also provided, as well as inheritance and transmission rates (F) of all SVs. In (F), child inheritance rate refers to the proportion of SVs in a child inherited from the parents. Parental transmission rate refers to the proportion of SVs in parents’ genomes that are transmitted and displayed here are all informative SVs that are only heterozygous in one parental genome. Vertical colored lines in each row represent the mean value, whereas numbers on the right margin represent median SV counts across the children or families. SV Classes: DEL, deletion; DUP, duplication; mCNV, multiallelic copy number variant; INS, insertion; INV, inversion; CPX, complex SV; CTX, inter-chromosomal translocation. Super-population ancestry labels: EUR, European; AFR, African; EAS, East Asian; SAS, South Asian; AMR, American. Descriptions of population labels are in Table S1. See also Figure S3.

We generated an SV call set across all 3,202 1kGP samples with short-read WGS data. These SV GTs were discovered and integrated from three analytic pipelines: GATK-SV (Collins et al., 2020), svtools (Abel et al., 2020), and Absinthe (Corvelo et al., 2021) (see STAR Methods) (Figure S3; Table S4). This final ensemble call set included 173,366 loci, composed of 90,259 DELs, 28,242 DUPs, 673 mCNVs, 49,693 INSs, 920 INVs, 3,568 complex SVs (CPXs), and 11 inter-chromosomal translocations (CTXs; Figure 2A; Table 1). The size and allele frequency distribution of SVs followed expectations; mobile element signatures were observed for ALU (200–300 bp), SVA (1–2 kb), and LINE1 (5–6 kb) variants (Figure 2B). Most SVs were rare, and SV allele frequencies were inversely correlated with SV size (Figure 2C). On average, ∼9,679 SVs were discovered in each genome (see Figure 2D; Table 1). The distribution of SVs observed per individual followed expectations for ancestry with the greatest number of SVs per genome derived from AFR populations (Figure 2E; Table 1) (Campbell and Tishkoff, 2008). The precision of the SV call set was also quite high, with a *de novo* SV rate of 3.5% (Figure 2F).

**Figure S4:**
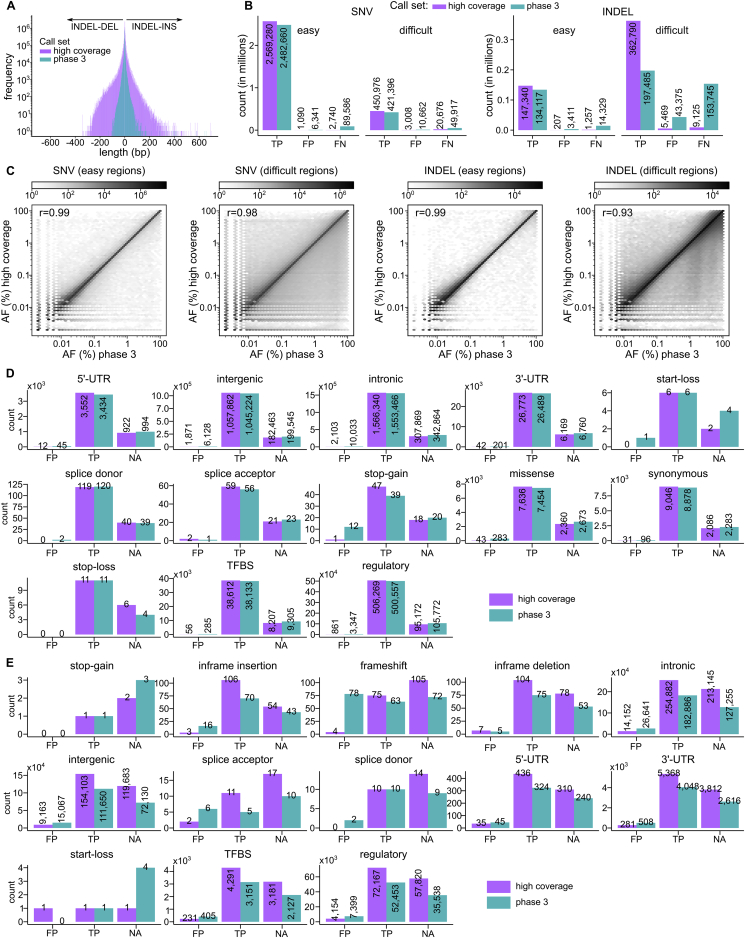
Comparison of small variant calls to the phase 3 call set, related to Figure 3 **(A)** Length of INDELs in the high-coverage as compared to the phase 3 call sets. **(B)** Number of true positive (TP), false positive (FP), and false negative (FN) SNVs and INDELs in the high-coverage vs. phase 3 call set, stratified by easy and difficult regions of the genome (GIAB v3.3.2 high confidence regions only). **(C)** Comparison of allele frequencies in the high-coverage vs. the phase 3 call set across shared loci, stratified by variant type and regions of the genome. r: Pearson correlation coefficient. Number of false positive (FP), true positive (TP), and unassessed (NA; sites outside of the GIAB v3.3.2 high confidence regions of the genome) predicted functional SNVs **(D)** and INDELs **(E)** in sample NA12878, defined based on the comparison against the GIAB NA12878 truth set v3.3.2. There were no stop-loss INDELs in sample NA12878 hence no plot for that category in E. See also Figures 3G and 3H (bottom row). Panels A, C, D, E: chr1-22; panel B: chr1-22 and X

### Comparison of the small variant calls to the 1kGP phase 3 call set

To quantify the improvements in the high-coverage resource, we compared our small variant calls against the original phase 3 call set. For consistency, we restricted this comparison to variants discovered in the original 2,504 samples (see STAR Methods).

**Figure 3:**
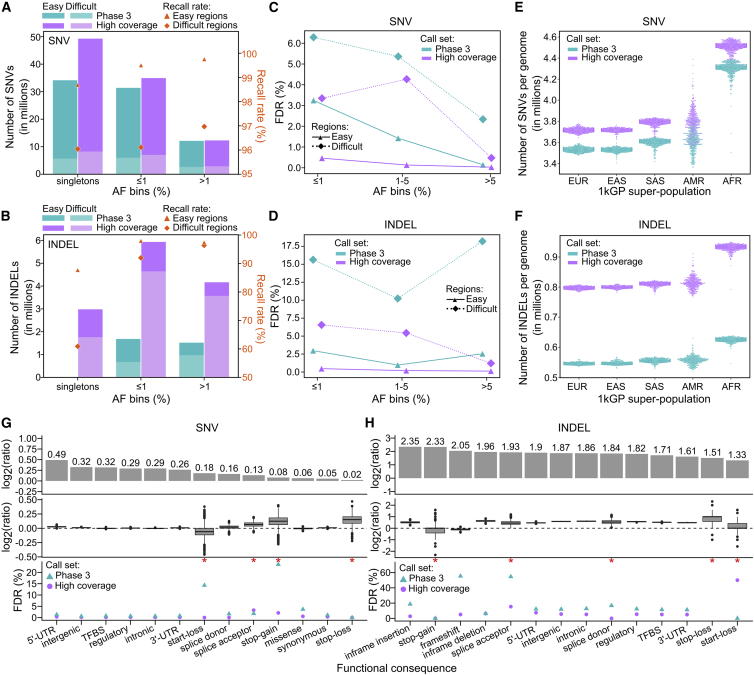
Comparison of small variant calls to the phase 3 call set (A and B) Number of SNVs (A) and INDELs (B) across the 2,504 samples in phase 3 and high-coverage datasets, stratified by AF bins and regions of the genome. Secondary y axis: % of autosomal phase 3 variants recalled in the high-coverage call set across SNVs (A) and INDELs (B) in easy and difficult regions of the genome. See also Figure S4C. (C and D) Comparison of FDR across SNVs (C) and INDELs (D) between the high-coverage and phase 3 call sets, stratified by AF bins and regions of the genome. See also Figure S4B. (E and F) Sample-level SNV (E) and INDEL (F) counts in the phase 3 versus high-coverage call sets, stratified by 1kGP super-population ancestry. EUR, European; AFR, African; EAS, East Asian; SAS, South Asian; AMR, American. Reported counts are at a locus level. (G and H) Comparison of predicted functional SNV (G) and INDEL (H) counts in the high-coverage versus phase 3 call set. Log2(ratio) denotes ratio of variant counts in the high-coverage versus phase 3 call set. Top row: cohort-level comparison. Middle row: sample-level comparison. Bottom row: comparison of FDR. Red asterisks mark categories with fewer than 100 sites in sample NA12878 (i.e., categories where FDR estimation is less reliable). See also Figures S4D and S4E. FDR in (C), (D), (G), and (H) was estimated based on comparison of calls in sample NA12878 to the GIAB truth set v3.3.2. (A), (B), and (E–H): chromosomes (chr) 1–22; (C) and (D): chr1–22 and X.

The 2,504-sample high-coverage call set includes 96,950,998 SNVs and 13,132,415 INDELs across the autosomes. This represents a 1.24-fold cohort-level increase in the number of SNVs and 4.05-fold increase in the number of INDELs compared to the phase 3 call set. Of the 10,322,838 INDELs that were called in the high-coverage call set but not in phase 3 (labeled henceforth as “new”), 60% were in homopolymer and tandem repeat regions (compared to only 10.5% of 22,455,268 new SNVs). The newly discovered INDELs are positioned across 6,511,277 distinct loci, a ratio of new INDEL alleles to new loci of 1.58, in comparison to INDELs that were previously discovered in phase 3, which are mostly biallelic with ratio of alleles to loci equal to 1.04. Among SNVs, we observed the largest gains in the number of singletons and rare alleles (AF ≤ 1%) in the high-coverage relative to the phase 3 call set. As expected, the number of common (AF > 1%) SNVs was similar across both call sets (Figure 3A). In the case of INDELs, we observed gains across the entire AF spectrum (Figure 3B). The highest increase (676-fold) was in the singleton category where the phase 3 call set contains only 4,437 singleton INDELs. The low number of ultra-rare INDEL calls in the phase 3 set can be attributed to more stringent filtering applied to INDELs as compared to biallelic SNVs (The 1000 Genomes Project Consortium, 2015) and limitations of low-coverage sequencing. The increase in the number of rare and common INDELs in the high-coverage versus phase 3 call set was also significant (Figure 3B). Additionally, we called significantly more INDELs above 50 bp in length (Figure S4A). At the per-sample level, we observed a 1.05-fold average increase in the number of SNVs and 1.47-fold increase in the number of INDELs in the high-coverage call set (Figures 3E and 3F).

Overall, we recalled 98.3% of the phase 3 small variants in the high-coverage call set with recall rate being higher in the easy versus difficult regions (Figures 3A and 3B). Shared variants displayed high correlation of AF between the high-coverage and phase 3 call sets (Figure S4C).

The FDR of the 2,504-sample high-coverage call set is 0.1% for SNVs and 1.1% for INDELs as compared to 0.6% for SNVs and 12.4% for INDELs in the phase 3 call set. In a stratified analysis, we observed significantly lower FDR across the entire AF spectrum, in both easy and difficult genomic regions, in the high-coverage as compared to the phase 3 call set (Figures 3C, 3D, and S4B). This trend was particularly pronounced among rare (AF ≤ 1%) SNVs and INDELs in the difficult regions of the genome.

We observed 1.01- to 1.40-fold increase in the number of SNVs falling into various functional categories in the high-coverage as compared to the phase 3 call set at the cohort level (Figure 3G). This increase was especially pronounced (≥1.2-fold) in the intronic, regulatory, transcription factor binding site (TFBS), intergenic, and untranslated region (UTR) variant categories. The rather insignificant increase in the number of SNVs in protein-coding categories (1.01- to 1.13-fold; Figure 3G) was expected since variant discovery in the phase 3 call set was based on high-coverage WES in addition to low-coverage WGS. We observed a more significant increase (2.5- to 5-fold) in the number of predicted functional INDELs in the high-coverage versus phase 3 call set at the cohort level (Figure 3H). At the sample level, the ratios of predicted functional SNV counts in the high-coverage versus phase 3 call set were close to 1 with well-controlled FDR in both call sets across nearly all categories. In the case of INDELs, the sample-level ratios were higher than for SNVs across most functional categories, reaching over 1.5 in case of inframe DELs as well as intergenic and intronic INDELs, consistent with a larger proportion of common INDELs relative to SNVs among new loci discovered in the high-coverage call set. We observed that relative differences in INDEL gains across coding versus non-coding categories are not as clear as in the case of SNVs. This is consistent with the fact that overall gains in INDEL calling in the high-coverage call set are not only due to increased coverage, as it is in case of SNVs, but also due to substantial improvements in calling algorithms, which apply to coding regions as well.

### Comparison of the SV calls to the 1kGP phase 3 call set

**Figure 4:**
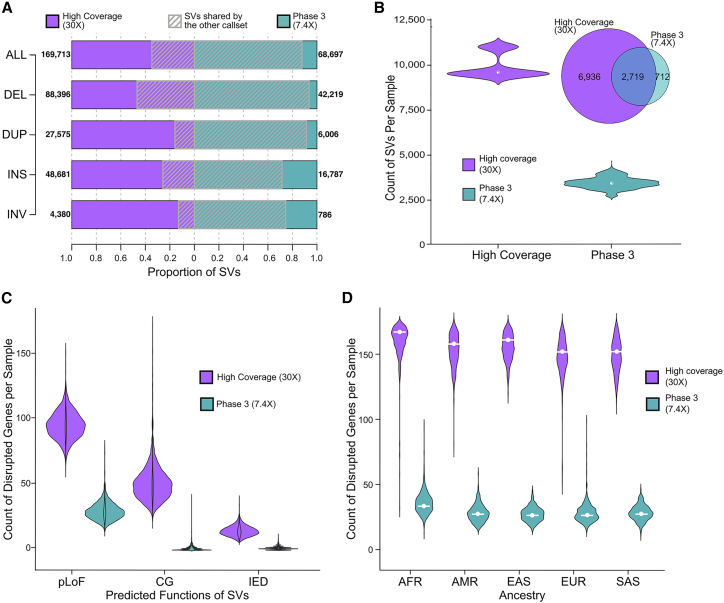
Comparison of the ensemble SV calls to the phase 3 call set (A) Count of SV sites in the current ensemble SV call set and phase 3 SV call set and their overlap. Numbers next to each bar represent the counts of SV sites in each dataset. (B) The distribution of SV counts per sample in both call sets and their average overlap, displayed in the Venn diagram. (C) Count of genes altered by SVs in both datasets. pLoF, predicted loss of function; CG, complete copy gain; IED, intragenic exon duplication. (D) Count of genes altered by SVs across ancestral populations. See also Figure S5.

The ensemble SV call set was compared to the 1kGP phase 3 SVs (Sudmant et al., 2015) on the 2,504 shared samples to assess improvements brought by high-coverage sequencing and genotyping capabilities of updated analytic pipelines. The current ensemble SV call set discovered over 2-fold more SV sites than phase 3 (169,713 versus 68,697) and encompassed 87.7% of the phase 3 SV calls (Figure 4A). This increased sensitivity and high overlap with phase 3 SVs was consistent across all SV classes (Figure 4A), with an average of 9,655 SVs detected per genome in the current ensemble call set compared to 3,431 SVs in the phase 3 call set (Figure 4B).

Precision of the high-coverage ensemble call set and 1kGP phase 3 calls was evaluated using long-read WGS on the 15 shared samples that had matched PacBio sequencing (Ebert et al., 2021; Sudmant et al., 2015). Two methods were applied for this evaluation: direct validation of SV through long reads using VaPoR (Zhao et al., 2017) and cross comparison of SV against assembly-based variants (Ebert et al., 2021). Results from both methods indicated comparable or higher precision across variant classes in the high-coverage ensemble calls compared to the phase 3 dataset (Table S5).

**Figure S5:**
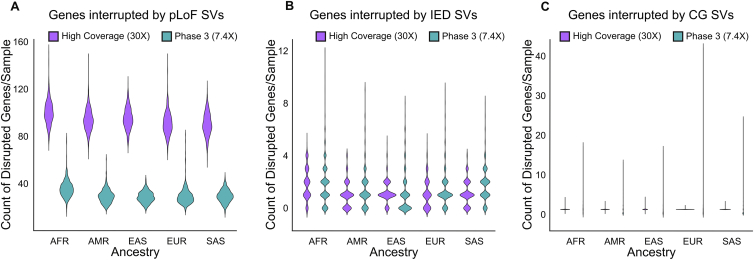
Comparison of gene interruptive SVs in the high-coverage ensemble versus phase 3 1kGP call sets, related to Figure 4 **(A)** Count of genes interrupted as predicted loss of function (pLoF), **(B)** intragenic exon duplications (IED), and **(C)** complete copy gain (CG) by SVs in the high-coverage ensemble call set and 1kGP phase 3 SV call set. Super-population ancestry labels: European (EUR), African (AFR), East Asian (EAS), South Asian (SAS), American (AMR)

The high-coverage SV call set provided significant added value in terms of the discovery of SVs that alter gene function by comparison to the phase 3 SV dataset. Consistent with a previous large-population study from short-read WGS that predicted disruption of 180 genes by SVs in each genome (Collins et al., 2020), as well as a recent study from the HGSVC using long-read WGS and complementary technologies that estimated 189 SVs per genome that altered protein coding genes (Ebert et al., 2021), we observed that biallelic SVs in each genome resulted in alteration of 162 genes per genome, including pLoFs of 97 protein coding genes, complete copy gain (CG) of 50 genes, and duplications of intragenic exons (IED) of 15 genes. Notably, the functional impact of IEDs has been previously shown to be correlated with negative selection against pLoF point mutations (Collins et al., 2020). This represents a considerable increase in the estimates from the phase 3 call set that predicted an average of 32 genes disrupted by SVs per genome (30 pLoFs, 1 CG, and 1 IEDs; Figures 4C and S5). The high-coverage 1kGP dataset also offered an estimate of the population diversity of functional SV variation, where AFR populations had most SVs per genome (Figure 4D), and similar patterns were observed when evaluating pLoF, CG, and IED SVs that altered protein coding sequences individually (Figure S5).

### Haplotype phasing

**Figure 5:**
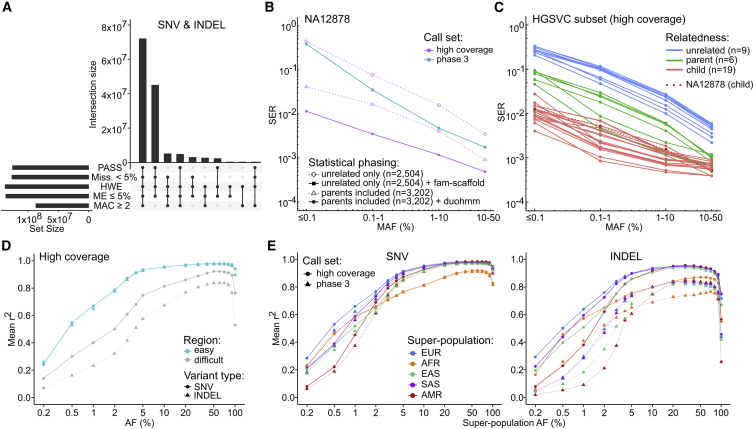
Small variant phasing and imputation performance (A) Counts of small variants passing specified filtering criteria (chr1–22 and X; top 10 combinations of filtering criteria in terms of variant counts are shown). PASS, sites that passed VQSR; Miss., genotype missingness; HWE, Hardy-Weinberg Equilibrium exact test p value > 1e-10 in at least one of the five 1kGP super-populations; ME, mendelian error rate across complete trios; MAC, minor allele count. See also Table S6. (B) Haplotype phasing accuracy of the high-coverage and the phase 3 1kGP panel. SER, switch error rate relative to the Platinum Genome truth set. Two additional phasing conditions (dashed lines) are shown for the high-coverage panel for evaluation purposes only: (1) diamonds: SER obtained when phasing NA12878 without parents included in the cohort. (2) Triangles: SER obtained when phasing NA12878 with parents included but without the pedigree-based correction (duohmm) applied. See also Figures S6A and S6B. (C) Haplotype phasing accuracy of the high-coverage panel, stratified by relationship status. SER was computed relative to the HGSVC SNV call set (Ebert et al., 2021). See also Figure S6C. (D) Imputation accuracy of SNV and INDEL genotypes imputed using the high-coverage panel, stratified by genomic regions. Mean r^2^, squared Pearson correlation coefficient averaged over 110 SGDP samples. See also Figures S6D–S6G. (E) Comparison of the imputation accuracy between the high-coverage and phase 3 panels for SNVs and INDELs, stratified by super-population ancestry. EUR, European; AFR, African; EAS, East Asian; SAS, South Asian; AMR, American. The comparison was restricted to sites that are shared between the two panels. (B–E) are based on autosomes.

**Figure 6:**
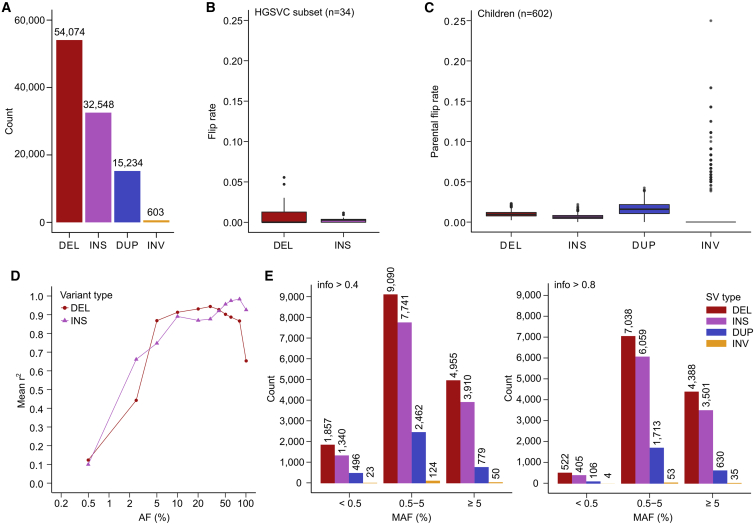
SV phasing and imputation performance (A) Cohort-level counts of filtered SVs included in the integrated haplotype-resolved panel, stratified by the SV type (chr1–22 and X). (B) Distribution of sample-level flip rate of phased HET DELs and INSs that were evaluated for phasing accuracy against the HGSVC truth set. (C) Distribution of sample-level parental flip rate of phased HET SVs, stratified by SV type. (D) SV imputation performance of the high-coverage panel in the SGDP study dataset, stratified by SV type. Mean r^2^, squared Pearson correlation coefficient between imputed allelic dosages and dosages from the SV “truth set,” averaged over the 110 SGDP samples (except for the AF = 0.5% bin: 100 and 92 samples for INSs and DELs, respectively). (E) Counts of SVs imputed in the SGDP study dataset using the high-coverage reference panel at info >0.4 (left) and info >0.8 (right) across three MAF bins (MAF based on 110 imputed SGDP samples). (B–E) are based on autosomes. SV types: DEL, deletions; INS, insertions; DUP, duplications; INV, inversions.

In addition to the small variant and SV call sets, we also generated an integrated haplotype-resolved SNV/INDEL/SV call set that can be used as a reference panel for imputation. We first performed haplotype phasing of high-quality non-singleton SNVs and INDELs (see Figure 5A and STAR Methods) across the 3,202-sample 1kGP cohort using statistical phasing with pedigree-based correction (see STAR Methods). Next, we used the phased SNV/INDEL call set as a haplotype scaffold onto which we phased high-quality non-singleton SV calls (Figure 6A). mCNV, CTX, and CPX SV types were excluded from the integrated reference panel due to being either ultra rare (CTX) or multiallelic and challenging to represent as distinct events for phasing (mCNV, CPX). The resulting integrated haplotype-resolved panel consists of 73,452,337 SNVs/INDELs and 102,459 SVs (DELs, INSs, DUPs, INVs) across autosomes and chromosome X (Figures 5 and 6A; Table S6).

**Figure S6:**
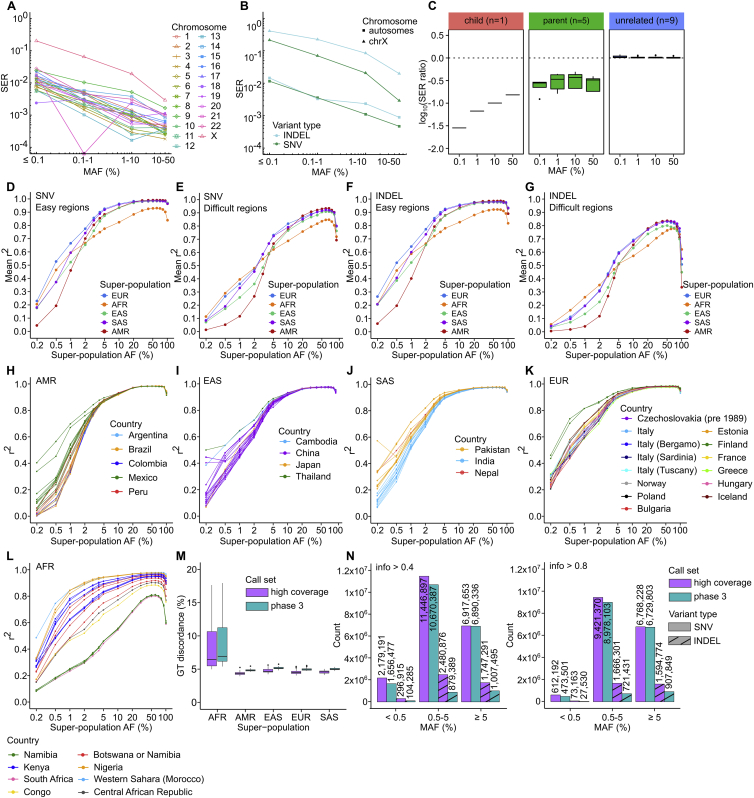
SNV/INDEL phasing and imputation performance, related to Figure 5 SER: switch error rate stratified by **(A)** chromosome and **(B)** variant type. Note: SER on chr21 in the 0.1–1% MAF bin is equal to 0 (i.e. no switch errors found). This is a fluctuation due to low variant counts per MAF bin in sample NA12878 as chromosomes get smaller. Chromosome X is shown separately in (B) as it was phased using a different strategy than autosomes (statistical phasing vs. statistical phasing with pedigree-based correction, respectively). **(C)** Impact of inclusion of trios on the phasing accuracy of the 1kGP high-coverage call set, stratified by relationship status in the 3,202-sample cohort. log10(SER ratio) refers to the ratio of SER in the phasing run including trios (n = 3,202 samples) vs. phasing run without trios (n = 2,504 samples), computed relative to the HGSVC truth set (1 child, 5 parents, 9 unrelated samples). Imputation accuracy of the high-coverage panel stratified by super-population for SNVs **(D, E)** and INDELs **(F, G)** in easy and difficult regions of the genome. Imputation accuracy was estimated as described in Figure 5D. **(H-L)** Imputation accuracy of the high-coverage panel for each of the five super-populations, stratified by the population. **(M)** Genotype discordance rates for SNVs and INDELs imputed using the high-coverage and phase 3 panels stratified by super-population. **(N)** Counts of SNVs and INDELs imputed in the SGDP study dataset using the high-coverage vs. the phase 3 reference panel at info >0.4 (left) and info >0.8 (right) across three MAF bins (MAF based on the 110 imputed SGDP samples). Panels C-N are based on autosomes

We evaluated phasing accuracy of the SNV/INDEL haplotype scaffold by computing switch error rate (SER) in sample NA12878 (child in the 1kGP cohort) relative to the gold standard phasing truth set, i.e. Platinum Genome NA12878 call set generated by Illumina (Eberle et al., 2017). The SER across all autosomes was 0.07% (across 2,338,955 assessed heterozygous [HET] SNV/INDEL pairs), indicating high accuracy of phasing. As expected, chromosome X displayed higher SER as compared to autosomes (SER = 0.49%, 73,794 HET pairs; Figure S6A). We did not observe a significant difference in phasing accuracy between SNVs and INDELs, other than on chromosome X (Figure S6B). We observed an expected increase in SER with decrease in MAF, but the SER remained low throughout the entire MAF spectrum, reaching a maximum of 1.14% in the ≤0.1% MAF bin across autosomes (Figure 5B).

To assess phasing accuracy of parental and unrelated samples in the haplotype scaffold, we used the haplotype-resolved call set from the HGSVC (Ebert et al., 2021), which includes phased SNV calls for 34 1kGP samples (19 children, 6 parents, 9 unrelated), as a phasing truth set. Based on this comparison and consistent with the expectation, phasing accuracy of children in the cohort is the highest (average autosomal SER = 0.09%) followed by parents (SER = 0.22%) and unrelated samples (SER = 0.79%) (Figure 5C). When compared against statistical phasing of just the 2,504 high-coverage unrelated samples, inclusion of trios (1) improved phasing accuracy of child samples from ∼35-fold in the ≤0.1% bin to ∼6.5-fold in the 10%–50% MAF bin, (2) improved phasing accuracy of parental samples ∼3- to 4.2-fold on average across MAF spectrum, and (3) had no significant effect on phasing accuracy of unrelated samples (Figure S6C).

We compared the phasing accuracy of the high-coverage SNV/INDEL haplotype scaffold to the phase 3 panel, which was phased using statistical phasing with family-based scaffold built from genotyping array data (The 1000 Genomes Project Consortium, 2015). The overall SER across autosomal SNVs and INDELs of the NA12878 sample in the phase 3 panel was 0.76% (2,238,400 HET pairs) relative to the Platinum Genome truth set, which is 10-fold higher than the corresponding SER in the high-coverage panel. The SER on chromosome X was 2.6-fold higher than the corresponding SER in the high-coverage panel. The significantly lower SER in the high-coverage as compared to the phase 3 panel was observed across all four MAF bins (Figure 5B), with magnitude of decrease ranging from 3.6-fold in the case of the most common MAF bin up to 34-fold in the rarest MAF bin. Phasing accuracy of the 2,504-sample phase 3 dataset was slightly better than that of the 2,504-sample high-coverage dataset (Figure 5B) because the latter dataset was phased using statistical phasing alone without the family-based scaffold. Compared to the 10-fold improvement in phasing accuracy of a child sample in the high-coverage versus phase 3 panel, parental and unrelated samples showed 2.2-fold and 1.3-fold average improvement, respectively, in the high-coverage panel across autosomes (relative to the HGSVC [Ebert et al., 2021] SNV phasing truth set).

**Figure S7:**
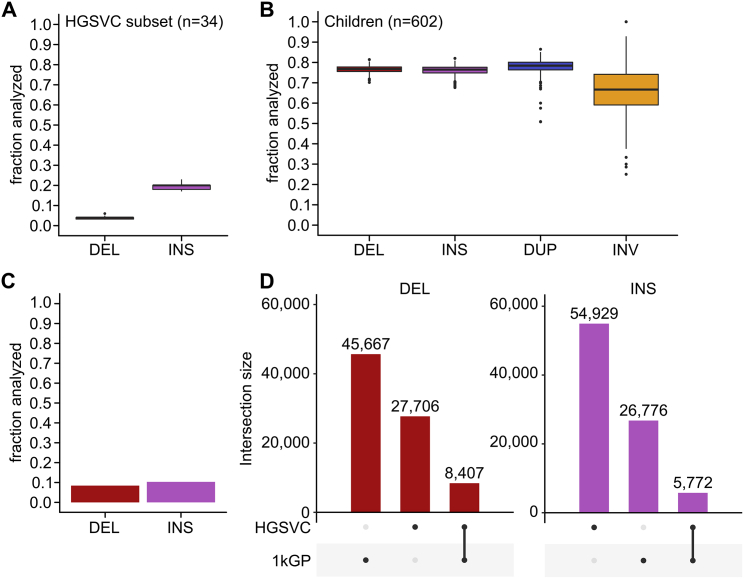
SV phasing and imputation performance, related to Figure 6 **(A)** Distribution of sample-level fractions of HET SVs (DELs and INSs) that were assessed for phasing accuracy against the HGSVC truth set in Figure 6B. **(B)** Distribution of sample-level fractions of HET SVs (DELs, INSs, DUPs, INVs) that were assessed for phasing accuracy using parental flip rate as shown in Figure 6C. **(C)** Fraction of SV sites (DELs and INSs; out of all DELs and INSs included in the high-coverage panel) that was included in the imputation performance evaluation against the HGSVC truth set shown in Figure 6D. **(D)** Upset plot showing site-level overlap of DELs and INSs discovered in the high-coverage 1kGP call set with those discovered in the long-read-based HGSVC call set used as the truth set. Overlap criteria: breakpoint position within +/−50 bp from the start site in the 1kGP call set and 80% length overlap. SV types: DEL: deletions, INS: insertions, DUP: duplications, INV: inversions

To evaluate phasing accuracy of SVs that we phased on top of the SNV/INDEL scaffold, we computed a fraction of SVs with flipped phase relative to the HGSVC call set (Ebert et al., 2021) across the 34 shared 1kGP samples (see STAR Methods). We restricted the analysis to DELs with 100% reciprocal overlap with the truth set (∼92 HET DELs per sample on average; ∼4% of total HET sites) and INSs with exactly matching breakpoint position (∼293 HET INSs per sample on average, ∼19% of total HET sites) (Figure S7A). Based on that, 0.24% of assessed INSs and 0.89% of assessed DELs had flipped phase per sample on average across the autosomes indicating high accuracy of phasing (Figure 6B).

As an orthogonal validation of SV phasing accuracy that (1) is independent of the truth set, (2) interrogates a higher fraction of HET sites, and (3) evaluates all four phased SV types, we computed the parental flip rate of phased heterozygous SV GTs across the 602 child samples (see STAR Methods). Using this strategy, we were able to assess phasing accuracy of ∼76%–78% HET DEL/INS/DUP (n = ∼1,392 DELs, ∼1,089 INSs, and ∼220 DUPs per sample on average) and ∼66% (n = ∼14) HET INV sites per child on average (Figure S7B). Based on that, the average parental flip rate of phased SVs across children was 0.65% for INSs, 0.99% for DELs, 1.63% for DUPs, and 1.20% for INVs across all autosomes (Figure 6C), providing further support for high accuracy of phasing across all SV types. For comparison, the average parental flip rate of phased HET SNVs/INDELs is 0.19% across autosomes.

### Imputation performance

We imputed a set of 110 diverse samples (Table S7) from the Simons Genome Diversity Project (SGDP) (Mallick et al., 2016) using the integrated high-coverage panel and evaluated the accuracy of imputed GTs by computing the squared Pearson correlation coefficient (r^2^) between imputed allelic dosages and dosages from WGS-based truth sets (see STAR Methods) across multiple AF bins. We observed significantly higher mean imputation accuracy in easy as compared to the difficult-to-sequence regions of the genome for both SNVs and INDELs imputed using the high-coverage panel (Figures 5D and S6D–S6G).

Next, we compared the SNV/INDEL imputation performance of the high-coverage panel to the phase 3 panel across shared loci. SNV imputation performance was comparable across the panels (Figure 5E). Imputation of INDELs with the high-coverage panel displayed superior accuracy across all five super-population ancestry groups across the entire AF spectrum (Figure 5E). SNV and INDEL GT discordance rates based on imputed dosages converted to hard-called GTs showed improved imputation performance with the high-coverage versus phase 3 panel (Figure S6M).

When evaluating SNV/INDEL imputation performance across all samples within a super-population ancestry group, we observe that accuracy can vary greatly depending on the specific ancestry of the sample (Figures S6H–S6L). Given that the 1kGP focused on ascertaining demographically large populations (Sudmant et al., 2015; The 1000 Genomes Project Consortium, 2015), diversity of the SGDP samples spanning 60 (as compared to 26 in 1kGP) different populations (Mallick et al., 2016) is not fully represented by the haplotypes available on the 1kGP panel, affecting imputation accuracy of certain populations, as seen in southern AFR samples (Figure S6L).

We evaluated counts of small variants imputed using both panels at two info score (metric of imputation confidence from the imputation software) thresholds across three MAF bins: very rare (MAF < 0.5%), rare (0.5 ≤ MAF < 5%), and common (MAF ≥ 5%). We observed that more variants were imputed at both info >0.4 and info >0.8 thresholds across all three MAF bins when using the high-coverage compared to the phase 3 panel (Figure S6N), with counts in the common MAF bin being most comparable, especially in case of SNVs. INDEL imputation with the high-coverage panel yielded higher counts across all MAF categories, even in the common bin in which we imputed ∼1.7-fold more INDELs compared to phase 3.

We separately evaluated imputation of the four SV types included in the integrated high-coverage panel (DELs, INSs, DUPs, INVs) into the SGDP samples. Similarly to what we observed for small variants (Figure S6N), 32% (n = 32,827) of SVs on the panel were imputed at info >0.4 (compared to 34% in case of SNVs/INDELs) and 24% (n = 24,454) of SVs were imputed at info >0.8 (compared to 27% in case of SNVs/INDELs) (Figure 6E). As expected, increasing the info threshold from 0.4 to 0.8 most substantially decreased the counts of very rare SVs. To evaluate the accuracy of imputed SV GTs, we first created an SV “truth set” by genotyping the catalog of SVs (DELs and INSs) from the HGSVC (Ebert et al., 2021) across the SGDP samples (see STAR Methods). Assessment of SV imputation accuracy (mean r^2^) across all SGDP samples was limited to only 10.3% (n = 3,253) and 8.4% (n = 4,320) of imputed INSs and DELs (Figure S7C), respectively, due to the rather small overlap between SVs discovered in the long-read-based HGSVC call set across 34 1kGP samples and those discovered in the short-read-based high-coverage 1kGP call set (Figure S7D). DELs and INSs showed comparable imputation accuracy to small variants at MAF > 5% (with INSs being comparable even at MAF > 2%) but lower accuracy at rarer MAF bins when evaluated against the HGSVC truth set (Figure 6D). While limited to a small evaluation set, these findings suggest that the high-coverage 1kGP SVs are accurately imputed, particularly at higher AFs, and that SV imputation performance follows the observed trend in the imputation of SNVs/INDELs at more common AF bins.

## Discussion

We present results from high-coverage WGS of the expanded 1kGP cohort, consisting of 2,504 original samples as well as additional 698 related samples, completing 602 trios in the cohort. We called 111,048,944 SNVs, 14,435,076 INDELs, and 173,366 SVs across the 3,202 samples using state-of-the-art methods. When compared to the low-coverage phase 3 1kGP dataset from 2015, the variant counts in the high-coverage call set reflect an estimated average increase of 190,885 SNVs (1.05-fold), 268,182 INDELs (1.47-fold), and 5,835 (2.81-fold) SVs per genome and a cohort-level increase of over 18.6 million SNVs (1.24-fold), 9.8 million INDELs (4.05-fold), and ∼100 thousand SVs (2.47-fold) across the original 2,504 unrelated samples. Our goal was not to dissect all the factors that likely influenced variant discovery in the high-coverage and phase 3 datasets but instead to provide an extensive assessment of gains that the technological advancements collectively brought to the high-coverage resource relative to phase 3.

As expected, given that the phase 3 dataset identified nearly all common SNVs (MAF > 1%) in the population, the vast majority of the SNVs identified here but not in phase 3 fall in the rare MAF spectrum (≤1%). Additionally, most new SNVs discovered here are non-coding as phase 3 included deeply sequenced WES in addition to low-coverage WGS data. Consistent with the fact that high-coverage sequencing and current variant callers bring greater improvements to INDEL as compared to SNV calling, we observed gains in INDEL counts across the entire MAF spectrum with gains in the rare end of the spectrum being the most pronounced.

The SVs presented here provide a significant increase in discovery power over the phase 3 call set. These data also have the benefit of extensive algorithm and variant assessment through a family-based design that permits evaluation of inheritance as well as scrutiny against orthogonal technologies. The *de novo* SV rate of 3.5% provided here is a reasonable, if imperfect, proxy for FDR. This proxy includes false positive SVs in the children, true *de novo* variants that are accurately predicted SVs (estimated to be ∼0.2%–0.5% from prior short-read WGS datasets [Collins et al., 2020; Turner et al., 2017; Werling et al., 2018]) and either false negative SVs in the parents or inaccurate breakpoint estimates in either the parent or child. It also includes somatic variants that arise in the cell lines over time, which we expect to be low by comparison to the above but nonetheless will contribute a fraction of variants to the *de novo* estimates. Notably, multiple properties of this SV call set, including SV counts, size and frequency distributions, and inheritance rates, are comparable to a previous study that utilized these methods on WGS from blood-derived samples and applied extensive molecular validation of *de novo* SV predictions (Werling et al., 2018). We performed manual inspection of all large CNVs (>50 kb, n = 4,180) and benchmarked large inversions against strand sequencing (Strand-seq) (>5 kb, n = 250) to assess orthogonal support. Notably, an important advance from the SV discovery in this dataset is the updated prediction of functional alterations from SVs in each human genome, which greatly exceeds estimates in the phase 3 call set (162 versus 32 genes altered per genome) and approaches predictions from long-read WGS datasets (∼189 genes altered; Ebert et al., 2021). The data presented here, coupled with the independent long-read WGS, Strand-seq, and optical mapping datasets on 34 of these samples from the HGSVC (Ebert et al., 2021), provide a valuable open access SV resource for methods development and genomic studies.

In addition to the SNV/INDEL and SV call sets, we also generated an improved haplotype-resolved reference imputation panel that can be used to impute high-quality SNVs, INDELs, and SVs into study datasets. Inclusion of 602 trios in the panel led to up to an order of magnitude greater accuracy of SNV/INDEL phasing relative to the phase 3 panel due to both an increase in long-range haplotype sharing between related samples and pedigree-based correction applied to child samples after statistical phasing to ensure consistency of phased haplotypes with the pedigree structure.

Most existing reference imputation panels, such as the HRC (The Haplotype Reference Consortium, 2016) and TOPMed (Taliun et al., 2021), do not yet include SVs due to challenges that SV calling and phasing present. Also, evaluation of SV phasing accuracy has been difficult so far due to unavailability of well-established haplotype-resolved SV truth sets, similar to Platinum Genomes (Eberle et al., 2017) or the GIAB (Wagner et al., 2022; Zook et al., 2019), that exist for SNVs/INDELs. The recently published SV call set from the HGSVC (Ebert et al., 2021) allowed us to circumvent the latter issue and provided a much needed reference for evaluation of SV phasing accuracy. Additionally, with the inclusion of trios in the expanded cohort, it is now possible to use inheritance patterns as an orthogonal way of validating phasing accuracy. Thanks to these developments, we were able to phase four SV types (DELs, INSs, DUPs, INVs) on top of the SNV/INDEL haplotype scaffold with high accuracy.

The lack of high-confidence genotyped SV call sets presented a challenge when attempting to evaluate the SV imputation performance of the high-coverage 1kGP panel. The fact that the HGSVC SV call set (Ebert et al., 2021) is both haplotype and sequence resolved (facilitated by long-read technology) enabled its use as a catalog of structural variation in the population for building an SV “truth set” in an independent SGDP study dataset. Evaluation of the SV imputation performance with such a truth set suggested high accuracy of imputed GTs, which was comparable to small variants, especially at MAF >5%.

For more than a decade, the 1kGP collection has been a key resource in the field of genomics. These datasets have produced scientific insights into population genetics and genome biology, as well as provided an openly sharable resource that has been widely used in methods development and testing as well as for technical validation. By generating high-coverage sequencing data for the complete phase 3 set of individuals and completing 602 trios with additional samples, we have updated this critical resource with benchmarks and standards for the next generation of large-scale international WGS initiatives. The higher coverage plus advances in sequencing and analytic methods greatly expanded the discovery of all rare variants and of INDELs and SVs across the frequency spectrum. The addition of many rare non-coding variants absent from the phase 3 set should enable different types of population genetic studies on the cohort. Our phased panel leveraging pedigree correction provides improvements in power across the board but particularly in the imputation of many more common INDELs and SVs, making these accessible through imputation for association studies. Importantly, this panel is fully public and can be freely downloaded and used in combination with other panels and for use with any target dataset. Although many larger sequencing projects have now been conducted, the open nature of the 1kGP samples will continue to make this a foundational resource for the community in the years to come.

### Limitations of the study

A direct comparison of the high-coverage 1kGP SNV/INDEL dataset to the phase 3 set was impossible due to differences in genomic reference builds that were used for variant calling during generation of the two call sets. To enable a locus-level comparison, we lifted-over the phase 3 dataset from the GRCh37 to the GRCh38 reference, which was successful for 99.9% of phase 3 SNVs/INDELs (the phase 3 SV call set is available on GRCh38). The assessment of FDR across SNVs/INDELs that fall within the difficult regions was limited as compared to the easy-to-sequence regions. This is because only 53.6% of difficult regions fall within the GIAB v3.3.2 high confidence regions of the genome compared to 91.1% of easy regions.

Consistent with previous analyses on a subset of these data (Zhao et al., 2021), SV discovery in short-read WGS displays limited sensitivity compared to assembly-based long-read methods in highly repetitive genomic regions, and this impact is most significant for insertions and SVs localized to simple repeats and segmental duplications (Ebert et al., 2021). Furthermore, we have not specifically included simple tandem repeats (STRs) in this SV call set, a subset of which can be captured in short-read sequencing though accurate genome-wide discovery that remains a considerable challenge (Dashnow et al., 2018; Dolzhenko et al., 2019; Mousavi et al., 2019).

The relatively small overlap of SVs between the 1kGP and the HGSVC (Ebert et al., 2021) call sets, due to limited ascertainment of SVs in short-read 1kGP data and inability to call low frequency variants across a small number of samples in the HGSVC long-read data, limited the number of SVs we could confidently evaluate from the high-coverage imputation panel. The SV truth set we built by genotyping the SGDP samples might be biased toward sites that are easier to GT and potentially easier to impute as it is composed of high-confidence GTs that were concordant between two SV genotypers.

In terms of the resource itself, the biggest limitation to consider is its LCL cell-line origin. We estimate that ∼5% of singletons in the high-coverage call set are truly present in the cell lines but may not represent true population variants in the original donors, which is important to consider when using the call set, e.g., as germline point of reference for cancer studies or as a resource for studying *de novo* mutations across the trios in the cohort.

## STAR★Methods

### Key resources table

**Table:** 

REAGENT or RESOURCE	SOURCE	IDENTIFIER
**Critical commercial assays**		

TruSeq DNA PCR-Free High Throughput Library Prep Kit	Illumina	Cat#20015963
KAPA Library Quantification Kits - Complete kit (Universal)	Roche	Cat#07960140001
HS NGS Fragment Kit	Agilent	Cat#DNF-474-0500
Quant-iT PicoGreen dsDNA Assay Kit	Life Technologies	Cat#P7589
IDT for Illumina – TruSeq DNA UD Indexes (Illumina, 20,022,370)	Illumina	Cat#20022370
SPRIselect Beads	Beckman Coulter	Cat#B23318
PhiX v3 Control	Illumina	Cat#FC-110-3001
NovaSeq 6000 S4 Reagent Kit (300 cycles)	Illumina	Cat#20012866
NovaSeq Xp Kit (4-lane)	Illumina	Cat#20021663

**Deposited data**		

raw sequence data FASTQ files	This paper	EMBL-EBI: PRJEB31736, EMBL-EBI: PRJEB36890
CRAM alignment files	This paper	EMBL-EBI: PRJEB31736, EMBL-EBI: PRJEB36890
CRAM alignment files	This paper	AnVIL: https://app.terra.bio/#workspaces/anvil-datastorage/1000G-high-coverage-2019/
CRAM alignment files	This paper	NCBI: https://ftp-trace.ncbi.nlm.nih.gov/1000genomes/ftp/1000G_2504_high_coverage/
CRAM alignment files	This paper	s3://1000genomes/1000G_2504_high_coverage/
GVCFs	This paper	IGSR: http://ftp.1000genomes.ebi.ac.uk/vol1/ftp/data_collections/1000G_2504_high_coverage/working/20190425_NYGC_GATK/raw_calls_updated/
SNV/INDEL VCFs	This paper	EMBL-EBI: PRJEB55077
SNV/INDEL VCFs	This paper	dbSNP: https://www.ncbi.nlm.nih.gov/SNP/snp_viewTable.cgi?handle=1000G_HIGH_COVERAGE (dbSNP: 1000G_HIGH_COVERAGE)
SNV/INDEL VCFs	This paper	IGSR: http://ftp.1000genomes.ebi.ac.uk/vol1/ftp/data_collections/1000G_2504_high_coverage/working/20201028_3202_raw_GT_with_annot/
SNV/INDEL VCFs (2,504-sample subset generated for evaluation purposes)	This paper	EMBL-EBI: PRJEB55077
SNV/INDEL VCFs (2,504-sample subset generated for evaluation purposes)	This paper	IGSR: http://ftp.1000genomes.ebi.ac.uk/vol1/ftp/data_collections/1000G_2504_high_coverage/working/20190425_NYGC_GATK/
Phased SNV/INDEL/SV VCFs	This paper	EMBL-EBI: PRJEB55077
Phased SNV/INDEL/SV VCFs	This paper	IGSR: http://ftp.1000genomes.ebi.ac.uk/vol1/ftp/data_collections/1000G_2504_high_coverage/working/20220422_3202_phased_SNV_INDEL_SV/
SV VCF	This paper	EMBL-EBI: PRJEB55077
SV VCF	This paper	IGSR: http://ftp.1000genomes.ebi.ac.uk/vol1/ftp/data_collections/1000G_2504_high_coverage/working/20210124.SV_Illumina_Integration/
SV VCF	This paper	dbVar: nstd206
lifted-over GRCh38 phase 3 1kGP SNV/INDEL VCFs	This paper	EMBL-EBI: PRJEB55077
lifted-over GRCh38 phase 3 1kGP SNV/INDEL VCFs	This paper	IGSR: http://ftp.1000genomes.ebi.ac.uk/vol1/ftp/data_collections/1000G_2504_high_coverage/working/phase3_liftover_nygc_dir/
Sample metadata file with pedigree and sex information	This paper	IGSR: http://ftp.1000genomes.ebi.ac.uk/vol1/ftp/data_collections/1000G_2504_high_coverage/working/1kGP.3202_samples.pedigree_info.txt

**Experimental models: Cell lines**		

genomic DNA from 3,202 samples from the 1000 Genomes Project	Coriell Institute for Medical Research	Data S1

**Software and algorithms**		

Absinthe	github.com/nygenome/absinthe	github.com/nygenome/absinthe
BCFtools v1.9, 1.12, and v1.15	Li (2011), Danecek et al. (2021)	http://samtools.github.io/bcftools/bcftools.html
BWA-MEM v0.7.15	Li (2013)	http://bio-bwa.sourceforge.net/
bedtools v2.26.0	Quinlan and Hall (2010)	https://github.com/arq5x/bedtools2
CrossMap v0.5.3	Zhao et al. (2014)	https://github.com/liguowang/CrossMap
Eagle v2.4.1	Loh et al. (2016)	https://alkesgroup.broadinstitute.org/Eagle/
FastQC v0.11.3	https://www.bioinformatics.babraham.ac.uk/projects/fastqc/	https://www.bioinformatics.babraham.ac.uk/projects/fastqc/
GATK v3.5 and v4.1	Van der Auwera and O'Connor (2020)	https://gatk.broadinstitute.org/hc/en-us
GATK-SV	Collins et al. (2020)	https://github.com/talkowski-lab/svtk
hap.py v0.3.12	github.com/Illumina/hap.py	github.com/Illumina/hap.py
IMPUTE v2.3.2	Howie et al. (2009)	https://mathgen.stats.ox.ac.uk/impute/impute_v2.html
KING v2.2.3	Manichaikul et al. (2010)	https://www.kingrelatedness.com/
PanGenie v1.0.0	Ebler et al. (2022)	https://github.com/eblerjana/pangenie
Paragraph v2.2b and v2.4a	Chen et al. (2019)	https://github.com/Illumina/paragraph
Picard v2.4.1	Van der Auwera and O'Connor (2020)	https://broadinstitute.github.io/picard/index.html
Plink v1.90 and v2.0	Chang et al. (2015)	https://www.cog-genomics.org/plink/1.9/
QCTOOL v2.0.2	https://www.well.ox.ac.uk/∼gav/qctool_v2	https://www.well.ox.ac.uk/∼gav/qctool_v2
R v3.6.1	https://www.r-project.org/	https://www.r-project.org/
RTG Tools v3.8.2	Cleary et al. (2015)	https://github.com/RealTimeGenomics/rtg-tools
Samtools v1.3.1	Li et al. (2009)	http://www.htslib.org/
SHAPEIT v2.r904	Delaneau et al. (2011)	https://mathgen.stats.ox.ac.uk/genetics_software/shapeit/shapeit.html
SHAPEIT4 v4.2.2	Delaneau et al. (2019)	https://odelaneau.github.io/shapeit4/
svtools	Larson et al. (2019)	https://github.com/hall-lab/svtools
Variant Effect Predictor (VEP) v104	McLaren et al. (2016)	https://useast.ensembl.org/info/docs/tools/vep/index.html
VCFtools v0.1.12	Danecek et al. (2011)	https://vcftools.github.io/index.html
VerifyBamID	Jun et al. (2012)	https://genome.sph.umich.edu/wiki/VerifyBamID
WhatsHap v0.18	Martin et al. (2016)	https://whatshap.readthedocs.io/en/latest/
LUMPY	Layer et al. (2014)	https://github.com/arq5x/lumpy-sv
Manta	Chen et al. (2016)	https://github.com/Illumina/manta
Wham	Kronenberg et al. (2015)	https://github.com/zeeev/wham
MELT	Gardner et al. (2017)	https://melt.igs.umaryland.edu/
cn.MOPS	Klambauer et al. (2012)	https://bioconductor.org/packages/release/bioc/html/cn.mops.html
CNVNator	Abyzov et al. (2011)	https://github.com/abyzovlab/CNVnator
GATK-gCNV	https://github.com/broadinstitute/gatk	https://github.com/broadinstitute/gatk
VaPoR	Zhao et al. (2017)	https://github.com/mills-lab/vapor

### Resource availability

#### Lead contact

Requests for further information and resources should be directed to and will be fulfilled by the lead contact, Michael Zody (mczody@nygenome.org).

#### Materials availability

This study did not generate new unique reagents.

### Experimental model and subject details

#### 1000 Genomes Project cohort

As part of this publication, we sequenced 3,202 lymphoblastoid cell line (LCL) samples from the 1kGP collection, including 1,598 males and 1,604 females. The 3,202 samples were drawn from 26 populations (listed in Table S1) across the following 5 continental ancestry groups: African (AFR, n = 893), European (EUR, n = 633), East Asian (EAS, n = 601), South Asian (SAS, n = 585), and American (AMR, n = 490) (Figures 1A and 1Table S1). Among the 3,202 samples, there are 602 father-mother-child trios (including 2 trios that are part of a multi-generational family, and 10 trios that were split from 5 quads for the purpose of pedigree-based correction applied after haplotype phasing) and 6 parent-child duos. All reported relationships were confirmed in IBD analysis using KING v2.2.3 (Manichaikul et al., 2010). All cell lines sequenced for this paper were obtained from Coriell Institute for Medical Research (NHGRI and NIGMS cell repositories). The following cell lines/DNA samples were obtained from the NIGMS Human Genetic Cell Repository at the Coriell Institute for Medical Research: [NA06984, NA06985, NA06986, NA06989, NA06991, NA06993, NA06994, NA06995, NA06997, NA07000, NA07014, NA07019, NA07022, NA07029, NA07031, NA07034, NA07037, NA07045, NA07048, NA07051, NA07055, NA07056, NA07340, NA07345, NA07346, NA07347, NA07348, NA07349, NA07357, NA07435, NA10830, NA10831, NA10835, NA10836, NA10837, NA10838, NA10839, NA10840, NA10842, NA10843, NA10845, NA10846, NA10847, NA10850, NA10851, NA10852, NA10853, NA10854, NA10855, NA10856, NA10857, NA10859, NA10860, NA10861, NA10863, NA10864, NA10865, NA11829, NA11830, NA11831, NA11832, NA11839, NA11840, NA11843, NA11881, NA11882, NA11891, NA11892, NA11893, NA11894, NA11917, NA11918, NA11919, NA11920, NA11930, NA11931, NA11932, NA11933, NA11992, NA11993, NA11994, NA11995, NA12003, NA12004, NA12005, NA12006, NA12043, NA12044, NA12045, NA12046, NA12056, NA12057, NA12058, NA12144, NA12145, NA12146, NA12154, NA12155, NA12156, NA12234, NA12239, NA12248, NA12249, NA12264, NA12272, NA12273, NA12274, NA12275, NA12282, NA12283, NA12286, NA12287, NA12329, NA12335, NA12336, NA12340, NA12341, NA12342, NA12343, NA12344, NA12347, NA12348, NA12375, NA12376, NA12383, NA12386, NA12399, NA12400, NA12413, NA12414, NA12485, NA12489, NA12546, NA12707, NA12708, NA12716, NA12717, NA12718, NA12739, NA12740, NA12748, NA12749, NA12750, NA12751, NA12752, NA12753, NA12760, NA12761, NA12762, NA12763, NA12766, NA12767, NA12775, NA12776, NA12777, NA12778, NA12801, NA12802, NA12812, NA12813, NA12814, NA12815, NA12817, NA12818, NA12827, NA12828, NA12829, NA12830, NA12832, NA12842, NA12843, NA12864, NA12865, NA12872, NA12873, NA12874, NA12875, NA12877, NA12878, NA12889, NA12890, NA12891, NA12892].

### Method details

#### WGS library preparation and sequencing

DNA extracted from LCLs was ordered from the Coriell Institute for Medical Research for each of the 3,202 1kGP samples. Whole-genome sequencing (WGS) libraries were prepared using the TruSeq DNA PCR-Free High Throughput Library Prep Kit in accordance with the manufacturer’s instructions. Briefly, 1ug of DNA was sheared using a Covaris LE220 sonicator (adaptive focused acoustics). DNA fragments underwent bead-based size selection (SPRIselect, Beckman Coulter) and were subsequently end-repaired, adenylated, and ligated to Illumina sequencing adapters (IDT for Illumina – TruSeq DNA UD Indexes (Illumina)). Final libraries were evaluated using fluorescent-based assays including measuring concentration with Quant-iT PicoGreen dsDNA Assay Kit (Life Technologies), qPCR with the Universal KAPA Library Quantification Kit and Fragment Analyzer (HS NGS Fragment Kit, Agilent) or BioAnalyzer (Agilent 2100). Libraries were sequenced on an Illumina NovaSeq 6000 system using 2 x 150bp cycles (NovaSeq 6000 S4 Reagent kit; NovaSeq Xp Kit; PhiX v3 Control (Illumina)).

#### Quality control of sequence data

We ran several quality control (QC) tools to look for quality issues, sample swaps, and contamination issues. We ran FastQC (Andrews, 2019) v0.11.3 on the raw sequence data to assess yield and raw base qualities. We ran Picard (Broad Institute, 2019) v2.4.1 CollectMultipleMetrics and CollectWGSMetrics on the aligned BAM to collect alignment and insert size metrics. Picard CollectGcBiasMetrics was run to compute normalized coverage across multiple GC bins. Read duplication metrics were quantified by running Picard MarkDuplicates on the BAM.

All the samples had at least 27X mean coverage across the genome (average per sample coverage: 34X, range: 27X-71X) and at least 91% of the bases at base quality score 30 or higher. The median insert size per sample was 433 bp. The mean duplicate rate across the samples was 9% but there were 5 samples (HG00619, HG00982, HG02151, HG02573 and HG04039) that had a duplicate rate greater than 20%. Higher duplication rate is a known issue with Illumina’s patterned flow cell that uses exclusion amplification clustering method to increase data output, but this chemistry is very sensitive to library loading concentrations. Higher loading concentrations can lead to low throughput because of polyclonal clusters being formed in the nanowells of the patterned flow cell, whereas low concentration can lead to pad hopping which increases the duplication rate. VerifyBamID (Jun et al., 2012) was run in chip-free mode to estimate the likelihood of sample contamination. None of the samples exceeded the 2% cut-off that we use for contamination (mean contamination across all the samples was 0.018% with a maximum of 1.36%).

To make sure there were no sample mix-ups we ran genotype concordance against genotyping chip data. For that, we used the chip data that was released with phase 3. We did not find chip data for 15 samples in phase 3 so for those we ran Infinium CoreExome-24 v1.3 chip and performed genotype concordance. All the samples had >97% genotype concordance.

#### SNV/INDEL discovery using GATK

Read alignment to the human reference genome GRCh38 using BWA-MEM v0.7.15 (Li, 2013), duplicate marking using Picard MarkDuplicates v2.4.1 (Broad Institute, 2019), and Base Quality Score Recalibration (BQSR) using GATK (McKenna et al., 2010) v3.5 BaseRecalibrator were performed according to the functional equivalence pipeline standard developed for the Centers for Common Disease Genomics project (Regier et al., 2018). SAM to BAM and BAM to CRAM file conversions were performed using Samtools v1.3.1 (Li et al., 2009). SNV and INDEL calling was performed using GATK (McKenna et al., 2010; Van der Auwera and O’Connor, 2020) v3.5, as described below. For variant discovery we used HaplotypeCaller in GVCF mode (Poplin et al., 2017) with sex-dependent ploidy settings on chromosome X and Y. Specifically, variant discovery on chromosome X was performed using diploid settings in females, diploid settings in pseudoautosomal (PAR) regions in males, and haploid settings in non-PAR regions in males. Variant discovery on chromosome Y was performed with haploid settings in males and was skipped entirely in females. We combined GVCFs in batches of ∼200 samples using GATK CombineGVCFs and jointly genotyped all 3,202 samples with GenotypeGVCFs. We then used VariantRecalibrator to train the Variant Quality Score Recalibration (VQSR) model using “maxGaussians 8” and “maxGaussians 4” parameters for SNVs and INDELs, respectively. We applied the VQSR model to the joint call set using ApplyRecalibration with truth sensitivity levels of 99.8% for SNVs and 99.0% for INDELs.

#### Evaluation of small variant calls

BCFtools v1.9 (Li, 2011) was used to split multiallelic variants into multiple rows and left-normalize INDELs before counting variants at the cohort level. Per sample variant metrics were collected using the GATK VariantEval tool (Van der Auwera and O’Connor, 2020). Mixed and complex variants and multi-nucleotide polymorphisms (MNPs) were not included in the breakdown of sample-level small variants. As part of QC, we estimated SNV density using the SNVDensity tool from VCFtools v0.1.12 (Danecek et al., 2011) in bins of 1000 bp across the callable genome, defined here as the GRCh38 reference excluding gaps (“N”s in the GRCh38 reference sequence). The mean SNV density across the callable genome is 39.46 per 1 kb of sequence (Table S2). Chromosome 19 (43.21 SNVs per 1 kb) has the highest density overall across all chromosomes, whereas Chromosome X (30.16 SNVs per 1 kb) displays the lowest density across all chromosomes, followed by chromosome 1 (36.46 SNVs per 1 kb) among the autosomes which is in agreement with previous reports based on WGS data (Telenti et al., 2016).

We evaluated small variant calls separately in easy- and difficult-to-sequence regions of the genome, using stratification intervals defined by the GIAB (Krusche et al., 2019) and obtained from https://ftp-trace.ncbi.nlm.nih.gov/giab/ftp/release/genome-stratifications/v2.0/GRCh38/union/.

Difficult regions include (i) tandem repeats and homopolymers longer than 6 bp (∼40% of difficult regions), (ii) segmental duplications (∼26% of difficult regions), (iii) low (<25%) and high (>65%) GC content regions and "bad promoters" (∼39% of difficult regions), and (iv) regions with low mappability (∼39% of difficult regions) with some overlap between categories (https://ftp-trace.ncbi.nlm.nih.gov/giab/ftp/release/genome-stratifications/v2.0/GRCh38/union/v2.0-GRCh38-Union-README.txt). Any region in the GRCh38 reference that did not fall into a difficult region was classified as easy. INDELs spanning any of the easy/difficult interval borders were classified as difficult. The easy and difficult regions make up 80 and 20% of the reference genome, respectively.

FDR was estimated both genome-wide and in easy vs. difficult regions of the genome by comparing variant calls in sample NA12878 from the 3,202-sample high-coverage call set to the GIAB NA12878 SNV/INDEL truth set v3.3.2 (Zook et al., 2019). The VCF files were compared using hap.py (v0.3.12; https://github.com/Illumina/hap.py) with the rtg-tools (v3.8.2) (Cleary et al., 2015) vcfeval comparison engine. All FDR calculations were restricted to the high confidence regions of the genome (consisting of 86.2% easy- and 13.8% difficult-to-sequence regions), as defined by the GIAB.

In addition to estimating FDR across all small variants and small variants in easy vs. difficult regions of the genome, we also estimated it among just the singletons. Due to the mixed nature of the expanded 1kGP cohort, which now includes both related and unrelated samples, the number of singletons (sites with AC = 1 across the 3,202 samples) per sample varies depending on the sample’s relatedness status, with children having the fewest singletons, followed by parents, and unrelated samples in the cohort (Figure S1E). We observed a nearly bimodal distribution of per-genome singleton counts among children with modes at 444 and 1,108 and the mean of 1,340, and a unimodal distribution among parents as well as unrelated samples with means at 12,365 and 23,197, respectively (Figure S1E). These differences are due to “private” variants (i.e. inherited variants that are private to a single family) which are not being counted as singletons in children, while 50 and 100% of them are being counted as singletons in each of the parents and in unrelated samples, respectively. In the high-coverage call set, each child in a trio carries on average 19,795 inherited autosomal heterozygous variants that are shared only with one or both parents across all samples in the cohort. These variants can be further broken down into sites with AC = 2 (mean = 19,658), AC = 3 (mean = 135), and AC = 4 (mean = 1.75) within a trio. The mean number of variants private to a family per child in a trio closely matches the difference between the mean per-genome singleton count in child vs. unrelated samples, in agreement with the expectation (Figure S1E). Approximately half of these ∼20,000 sites are shared between the child and the mother and the other half between the child and the father, hence the mean singleton count in parents is halfway in between the mean singleton count in child and unrelated samples. Since NA12878 is a child in the expanded 1kGP cohort, we jointly assessed both its *de novo* variants (n = 2,404) as well as inherited heterozygous variants that are private to the NA12878 trio (n = 15,131) to estimate FDR among singletons. To ensure that this approach for FDR estimation is not biased due to inclusion of NA12878’s parents in joint genotyping, we also computed FDR among singletons in NA12878 from an independent jointly genotyped high-coverage call set consisting of just the original 2,504 1kGP unrelated samples. Both of the FDR singleton analyses were restricted to the high confidence regions of the genome, as defined by either the GIAB v3.3.2 (Zook et al., 2019) or GIAB v4.2.1 (Wagner et al., 2022) truth sets.

Counts of assessed singletons in both FDR analyses

**Table:** 

Source of the evaluated NA12878 variant calls	Total count	# Evaluated sites w/in the GIAB v3.3.2 high confidence regions	# Evaluated sites w/in the GIAB v4.2.1 high confidence regions
High-coverage 3,202-sample joint GT-ing	2,404 DNMs +15,131 private variants	1,348 DNMs +12,737 private variants	967 DNMs + 13,696 private variants
High-coverage 2,504-sample joint GT-ing	16,837 singletons	13,876 singletons	14,354 singletons

#### Functional consequence of small variants

We annotated small variant calls with predicted functional consequence using the Ensembl Variant Effect Predictor (VEP) v104 tool (McLaren et al., 2016). For each site, we chose one functional consequence per allele-gene combination (using “--pick_allele_gene” parameter) with default ordering of selection criteria. To avoid bias coming from families and to facilitate comparison to the phase 3 call set, cohort- and sample-level counts per predicted functional categories were reported based on the 2,504-sample jointly genotyped high-coverage call set which includes unrelated samples only (see Methods subsection below). Only variants that passed VQSR, had GT missingness rate of <5%, and were in Hardy-Weinberg Equilibrium (HWE exact test p value > 1e-10 in at least one of the five super-populations) were considered in summary counts. No other filtering criteria were applied unless specifically noted.

#### Comparison of SNV/INDELs to the phase 3 set

To enable comparison of the high-coverage against the phase 3 call set, we lifted-over the SNV/INDEL calls in the phase 3 call set from the GRCh37 to GRCh38 reference build using CrossMap v0.5.3 (Zhao et al., 2014). As input to the lift-over, we used the phase 3 VCFs available on the 1000 Genomes FTP, http://ftp.1000genomes.ebi.ac.uk/vol1/ftp/release/20130502/. Prior to the lift-over, we split multiallelic sites into separate rows. A small fraction of phase 3 loci (0.1%) failed the lift-over step due to the following reasons: 1) no hit found (unmapped GRCh37 variants); 2) loci mapping to multiple locations in the GRCh38 (multiple hits); 3) the reference allele matches the alternate allele after the lift-over (REF = ALT allele in the GRCh38). Additionally, we excluded variants that were lifted-over to a chromosome that was different from the original chromosome in GRCh37 (chromosome mismatch) or if the reference allele contained non-canonical nucleotide bases (non-canonical REF). Using this approach we were able to successfully lift-over 99.9% of phase 3 small variant loci (see table below). The resulting GRCh38 phase 3 call set that was used for the comparison was restricted to autosomes and contained 78,324,761 SNVs and 3,244,241 INDELs.

Table summarizing lift-over failures in the small variant phase 3 call set, consisting of 81,646,103 SNV/INDELs total

**Table:** 

Failure reason	Count
REF = ALT allele in the GRCh38	27,889
Unmapped GRCh37 variants	15,856
Multiple hits	420
Chromosome mismatch	32,919
Non-canonical REF	17
**Total lift-over failures**	**77,101 (0.1%)**

We restricted the comparison of the high-coverage vs. phase 3 calls to the 2,504 samples in common to the two cohorts. For that purpose, we generated an independent jointly genotyped high-coverage call set, including only the 2,504 original samples (deposited in EMBL-EBI and IGSR FTP, see key resources table). Difference in FDR estimation between the 2,504- vs. 3,202-sample high-coverage call set (0.1% vs. 0.3% for SNVs, respectively) is due to between-run variability caused by the non-deterministic nature of the VQSR step of the GATK SNV/INDEL calling pipeline (number of false positive SNVs across VQSR PASS sites: 4,098 vs. 9,227; number of false positive SNVs across all called sites: 22,807 vs. 22,994, in the 2,504- vs. 3,202-sample joint genotyping, respectively). The comparison of high-coverage vs. phase 3 small variant call set was restricted to autosomes only. AF correlation across SNV and INDEL sites that are shared between the high-coverage and the phase 3 call set was calculated using Pearson correlation coefficient obtained using the cor() function in R.

To compare the counts of small variants per functional consequence category between the high-coverage and phase 3 call set, we annotated the GRCh38 lifted-over version of the phase 3 call set with the Ensembl VEP (the same way as described for the high-coverage call set above), and computed ratios of cohort- and sample-level counts in the high-coverage call set vs. phase 3 call set (filtered using the same criteria as described for the high-coverage call set above). To assess FDR across SNVs and INDELs in each functional category, we compared predicted functional SNVs and INDELs in the high-coverage and phase 3 call sets to the GIAB NA12878 truth set v3.3.2 (Zook et al., 2019). The FDR calculation was restricted to the high confidence regions of the genome, as defined by the GIAB.

#### SV discovery using GATK-SV

GATK-SV involved an ensemble SV discovery and refinement pipeline for WGS data. The technical details of the method were previously described in Collins et al. (Collins et al., 2020) for application to the genome aggregation database (gnomAD) for SV discovery, and further described in analyses from the HGSVC (Ebert et al., 2021). In this study, the same methods were applied to all 3,202 samples for SV discovery. In brief, SVs discovered by Manta (Chen et al., 2016), Wham (Kronenberg et al., 2015), MELT (Gardner et al., 2017), cn.MOPS (Klambauer et al., 2012), and GATK-gCNV (https://github.com/broadinstitute/gatk) were integrated, genotyped across all samples, resolved for complex SVs, and annotated for variant class and functional impact. The FDR was previously assessed from analyses in quartet families, which yielded a 97% molecular validation rate for *de novo* SV predictions (Werling et al., 2018), as well as a 94% validation rate compared to long-read sequencing (Collins et al., 2020).

#### SV discovery using svtools

The svtools (Larson et al., 2019) method was previously described in (Abel et al., 2020) and applied for SV discovery across 17,795 genomes from the Centers for Common Disease Genomics (CCDG) program (Abel et al., 2020). The workflow combines per-sample variant discovery with lumpy (Layer et al., 2014) and manta (Chen et al., 2016) with resolution-aware cross-sample merging. The set of merged variants is then genotyped with svtyper (Chiang et al., 2015), followed by copy-number annotation with CNVnator (Abyzov et al., 2011) and reclassification of variants based on concordance of read-depth with breakpoint orientation. All parameter settings and versions are as implemented in the wdl-based workflow (https://github.com/hall-lab/sv-pipeline).

#### Large insertion discovery using Absinthe

On a per-sample basis, insertions with a minimum length of 100bp were discovered through *de novo* assembly of unmapped and discordant read pairs using Absinthe (Corvelo et al., 2021), and then genotyped using Paragraph (Chen et al., 2019), respecting sex-specific ploidies. Insertion calls from all 3,202 samples that were positively genotyped with a PASS filter flag were then clustered by genomic location and aligned using MAFFT (Katoh and Standley, 2013). For each locus, the most consensual allele was selected. Variants from the resulting merged call set were then re-genotyped with Paragraph v2.2b (Chen et al., 2019) on all 3,202 individuals. To produce the final call set only variants with 1) genotyping PASS filter rate ≥80%; 2) Mendelian Error Rate ≤5% for complete trio calls; and 3) HWE Chi-square test p value > 1 × 10^−6^ in at least one of the 5 super-populations were kept.

#### Integration of SV call sets

We conducted a series of analyses to benchmark SVs from each of the three methods described above, including their FDR as indicated by inheritance rates and support from orthogonal technologies, as well as their breakpoint precision estimated by the deviation of their SV breakpoints from long-read assemblies in three genomes from analyses in the HGSVC (Chaisson et al., 2019). We also compared the three call sets to decide on the optimal integration strategy to maximize sensitivity and minimize FDR in the final ensemble call set (Figure S3, Table S5). Details of the comparison and integration strategies are described separately for insertions and all other variant classes below.

#### Integration of insertions

We compared the *de novo* rate of variant calls from each pipeline for insertions, yielding results of 4.1% for GATK-SV, 25.8% for svtools, and 2.4% for Absinthe. Given these results we restricted integration of insertions to GATK-SV and Absinthe. Each insertion pair was considered concordant if the insertion points were within 100 bp. The FDR of each insertion call set was estimated from three measurements: 1) *de novo* rate of SVs observed in the 602 trios; 2) proportion of SVs that were not validated by VaPoR (Zhao et al., 2017), an algorithm that evaluates SV quality by directly comparing raw PacBio reads against the reference genome, and 3) proportion of SVs that were not overlapped by SVs from PacBio assemblies in the same genome (Figure S3D). Precision of an insertion call was estimated by the distance of the insertion point to the closest PacBio insertion and the difference between the length of inserted sequence versus the length of the closest PacBio insertion calculated as an odds ratio. Both insertion call sets display less than 5% FDR based on inheritance and PacBio support, and the call sets were thus merged for all subsequent analyses (Figure S3D). Notably, as Absinthe showed higher precision than GATK-SV, as measured from both the coordinates of the insertion point and the length of inserted sequences (Figure S3H, I), we retained the Absinthe record for insertions that were shared by both methods.

#### Integration of SVs other than insertions

To consider a pair of SVs of the same variant class other than insertions as concordant, 50% reciprocal overlap was required for SVs larger than 5 kb and 10% reciprocal overlap was required for variants under 5 kb. The FDR across variant calls was evaluated using the same measurements as described above. For deletions, duplications, and inversions, we observed low FDR (<5%) among variants that were shared by GATK-SV and svtools, but significantly higher FDR in the subsets that were uniquely discovered by either algorithm (Figure S3E-G). To restrict the final call set to high-quality variants, a machine learning model (lightGBM (Ke et al., 2017)) was trained on each SV class. Three samples that were previously analyzed in the HGSVC studies (HG00514, HG00733, NA19240) (Chaisson et al., 2019; Ebert et al., 2021) were selected to train the model. The truth data was defined by SVs that were uni-parentally inherited, shared by GATK-SV and svtools, supported by VaPoR, and overlapped by PacBio call sets. The false training subset was selected as SVs that appeared as *de novo* in offspring genomes, specifically discovered by either GATK-SV or svtools, not supported by VaPoR, and not overlapped by PacBio call sets. Multiple features were included in the model, including the sequencing depth of each SV, the depth of the 1 kb region around each SV, the count of aberrant pair ends (PE) within 150 bp of each SV, the count of split reads (SR) within 100 bp of each breakpoints, the size, allele fraction and genomic location (split into short repeats, segmental duplications, all remaining repeat masked regions, and the remaining unique sequences) of each SV, and the fraction of offspring harboring a *de novo* variant among trios in which the SV is observed. Each SV per genome was assigned a ‘boost score’ by the lightGBM model, and SVs with >0.448 boost score were labeled as ‘PASS’ in the model (Figure S3M, S3N). This threshold was specifically selected to retain an estimated FDR <5%. Call set specific SVs that failed the lightGBM model in less than 48% of all examined samples were included in the final integrated call set (Figure S3N).

To design strategies to merge SVs shared by GATK-SV and svtools, the precision of SV calls was evaluated by examining the distance between breakpoint coordinates of SVs to matched calls in the PacBio call set. Comparable breakpoint precision was observed for GATK-SV and svtools (Figure S3J-L). Thus, for SVs in each sample, the variant with the greatest number of split reads for each breakpoint was selected, or if equivalent then the variant with the higher boost score was retained, then for each locus the SV observed in the greatest number of samples was retained as final.

#### Inclusion of SVs exclusively from GATK-SV

Other minority SVs types, including mCNVs, CPX and CTX, were specifically detected by GATK-SV, so we performed in-depth manual inspection to ensure their quality before including them in the final integration call set. The depth profile across all 3,202 samples around each mCNV was plotted for manual review, and mCNVs that did not show clear stratification among samples were labeled as ‘Manual_LQ’ in the filter column even if they showed clear deviation from the normal copy number of 2. For CTX, the aberrantly aligned read-pairs across each breakpoint were manually examined, and variants that lacked sufficient support were labeled as ‘Manual_LQ’ in the final call set.

#### Comparison of SVs to the phase 3 call set

We compared the quality of SVs from the high-coverage WGS to the 1kGP phase 3 SV call set reported by Sudmant et al. (2015). Phase 3 SVs aligned against GRCh38 were obtained from the 1kGP ftp site: http://ftp.1000genomes.ebi.ac.uk/vol1/ftp/phase3/integrated_sv_map/supporting/GRCh38_positions/. It should be noted that 121 SVs failed lift-over and were removed from the GRCh38 VCF, so a total of 68,697 SV sites were included in this comparison instead of 68,818 which was reported in Sudmant et al. (2015). When comparing SVs, we required 10% or higher reciprocal overlap for CNVs and INVs under 5 kb to be considered concordant, and 50% or higher reciprocal overlap for CNVs and INVs that are over 5 kb. We consider insertion pairs with insertion point within 100bp as concordant.

#### Generation of SNV/INDEL haplotype scaffold

To filter the SNV/INDEL call set for haplotype phasing, we first annotated the call set with HWE exact test p values (Wigginton et al., 2005), stratified by super-population, using the BCFtools v1.9 *fill-tags* plugin (Li, 2011). Next, we split multiallelic sites into separate rows and left-normalized representation of INDELs using BCFtools *norm* tool (Li, 2011). To ensure distinct start position of all variant loci, required for phasing, we shifted positions of multiallelic sites by a minimum possible number of bp using an in-house script. The positions were shifted back to the original ones after phasing. The following criteria were used to filter SNVs and INDELs for phasing: FILTER (column in the VCF) = PASS, GT missingness rate <5%, HWE exact test p value > 1e-10 in at least one super-population, mendelian error rate (MER) ≤ 5%, and minor allele count (MAC) ≥ 2 (singletons were removed because they are not informative for phasing). Filtering was done using BCFtools v1.9 (Li, 2011), except for MER filtration which was done using plink v1.90 (Chang et al., 2015) after VCF to plink conversion (required to run phasing). For VCF to plink conversion we used plink v2.0 (Chang et al., 2015). For haplotype phasing we used statistical phasing with pedigree-based correction, as implemented in SHAPEIT-duohmm v2.r904 (Delaneau et al., 2011; O’Connell et al., 2014). Phasing with SHAPEIT-duohmm was performed per chromosome using default settings, except for the window size parameter "-W'' which was increased from 2Mb (default) to 5Mb to account for increased amounts of shared IBD due to pedigrees being present in the dataset (as recommended in the SHAPEIT manual). SHAPEIT-duohmm supports phasing of autosomal variants only. Therefore, to phase variants on chromosome X, we used statistical phasing as implemented in the Eagle v2.4.1 software (Loh et al., 2016). Phasing with Eagle was performed using default parameters. No shifting of positions for multiallelic sites was needed as Eagle supports phasing of variants with the same start site. SHAPEIT set a small fraction of rare sites (116,417 SNVs and 57,655 INDELs; 0.24% small sites total) into sites with MAC <2 (monomorphic or singleton) during phasing. We removed these sites from the haplotype scaffold by running another round of MAC ≥2 filtering using BCFtools v1.9 which resulted in 73,452,337 small variants (63,993,320 SNVs and 9,459,017 INDELs) in the final panel. Phasing accuracy evaluation was performed using the WhatsHap tool v0.18 (Martin et al., 2016). As a measure of phasing accuracy we used switch error rate (SER), which is defined as:

$$SER=\frac{number\;of\;switch\;errors}{number\;of\;assessed\;HET\;pairs}$$

In all of the SNV/INDEL phasing evaluations, SER was computed across pairs of consecutive heterozygous sites either in sample NA12878 (child in a trio in the expanded 3,202-sample cohort) relative to the Platinum Genome NA12878 gold standard truth set (Eberle et al., 2017) or in a subset of 34 1kGP samples included in the HGSVC call set (Ebert et al., 2021) which we used as a phasing truth set to expand evaluations of phasing accuracy to samples across all relationship types present in the cohort.

#### Phasing of SVs

SV calls were filtered using the same criteria as described above for SNVs and INDELs. The filtered SV VCF was integrated with the phased SNV/INDEL haplotype scaffold VCF using BCFtools v1.12 concat with “--allow-overlaps” option (Danecek et al., 2021). Four types of SVs (DEL, INS, DUP, and INV) were phased on top of the SNV/INDEL haplotype scaffold using SHAPEIT4 v4.2.2 (Delaneau et al., 2019) with “--scaffold” and “--sequencing” options. mCNV, CTX, and CPX SV types were excluded from phasing due to being either ultra rare (CTX) or multiallelic and challenging to represent as distinct events for phasing (mCNV, CPX). SHAPEIT4 produced diploid output across the entire chromosome X in all samples. To ensure proper ploidy of male samples in the phased panel, we converted “0|1”, “1|0”, and “1|1” GTs into a haploid representation (i.e. “1”) in non-PAR regions of chromosome X in males. The strategy we adopted for evaluation of phasing accuracy of SVs is based on the following considerations: 1) since there are a lot more SNV/INDELs than SVs in the genome, analysis of SER genome-wide across all variant types would be dominated by SNV/INDELs and would not be informative for evaluating accuracy of SV phasing; 2) since distances between SVs along the genome are significantly greater than between SNV/INDELs, assessment of SER across pairs of consecutive HET SVs (analogous to how one does assessment of SNV/INDEL phasing) is by definition biased to produce significantly higher SER for SVs than for SNVs. This is because the greater the distance between two assessed HET sites the greater the chance of encountering a switch error. While the haplotype scaffold exhibits low switch error rate (∼0.09% across child haplotypes, ∼0.22% across parental haplotypes, and ∼0.89% across unrelated sample haplotypes), there are ∼1,358 small variants between every pair of DELs and ∼2,257 small variants between every pair of INS, on average (given 73,452,337 small variants, 54,074 DELs, and 32,548 INS in the panel). These numbers get even larger if we restrict the analysis only to HET SVs present in the truth set. This suggests that even at the lowest average SER of 0.09% observed across child haplotypes, we expect for there to be at least one switch error between any two DELs or INSs in the scaffold itself, statistically speaking. Considering the two factors outlined above, we assessed phasing accuracy of SVs using two orthogonal approaches. In the first one, we computed the flip rate only for pairs of SVs and their flanking SNVs on both sides. We defined flip rate as the fraction of SVs that have flipped phase relative to the SV–flanking-SNV pairs from the HGSVC call set (Ebert et al., 2021) which we used as the truth set. To avoid issues stemming from improper matching of SVs between the evaluation call set and the truth set, we restricted the analysis to DELs with 100% reciprocal overlap with the truth set and INSs with exactly matching breakpoint position. In the second approach, which is independent of the truth set, we computed the parental flip rate of phased SV GTs across the 602 child samples in the cohort for each of the four SV types. We define parental flip rate as a fraction of phased GTs in a child sample that are inconsistent with inheritance patterns based on the comparison to parents, considering only sites with unambiguous trio phase (excluding sites where child, father, and mother are all HET and sites with *de novos*).

#### Imputation performance evaluation

To evaluate the imputation performance of the high-coverage reference panel, we imputed 110 samples from 60 diverse populations, listed in Table S7, from the Simons Genome Diversity Project (SGDP) (Mallick et al., 2016). To create a pseudo-GWAS input dataset from the SGDP WGS data, we extracted genotypes at all sites included on the Illumina Infinium Omni2.5-8 v1.4 array from the jointly genotyped SNV/INDEL SGDP call set generated using the GATK Best Practices workflow (Poplin et al., 2017). We performed quality control (QC) of the dataset using standard pre-imputation filters, removing sites which did not meet the following criteria: genotype call rate of ≥95%, MAF >1%, and HWE (exact test p value ≥ 1 × 10^−4^). We used plink v1.9 (Chang et al., 2015) for all QC steps, and analysis was restricted to the autosomes. We imputed the data passing quality control with the phase 3 and the high-coverage reference panels, separately. We used the lifted-over GRCh38 phase 3 call set (described above) for all phase 3 panel evaluations and excluded any monomorphic or singleton sites for consistency with the high-coverage panel, resulting in 47,016,818 SNV/INDEL sites in the phase 3 imputation panel. The high-coverage panel contains 73,452,337 SNV/INDEL and 102,459 SV sites. Before imputation, we used SHAPEIT v2.r904 (Delaneau et al., 2011) to perform a strand check and remove any problematic sites as determined by aligning with the respective panel. Pre-phasing was also performed using SHAPEIT and the reference panel used for imputation (either the phase 3 or the high-coverage panel). We then imputed the pre-phased data using IMPUTE v2.3.2 (Howie et al., 2009) software with default parameters. Following imputation, we concatenated the imputed intervals to create an autosome-wide imputed dataset. To assess how confidently we are imputing variants in the SGDP study set, we used metrics calculated on this dataset, IMPUTE reported MAF and info values (which are estimated using expected AF in the imputed set), to determine counts of imputed variants across various MAF thresholds and info cutoffs (Figures S6N, 6E). We evaluated imputation using all 110 samples with 22 samples from each of the five super-population ancestry groups (EUR, AFR, SAS, EAS, and AMR), the maximum number of samples available across all populations, and compared imputed dosages with truth set dosages stratified by AF (calculated using allele counts of unrelated samples from the high-coverage panel; Figures 5D-E, S6D-L, 6D). For the SNV/INDEL imputation accuracy, we used the jointly genotyped WGS SGDP data, restricted to polymorphic sites, as the truth set. The SV truth set used for evaluations is described below (see “Generation of the SV truth set”). We converted the imputed posterior genotype probabilities produced by IMPUTE v2.3.2 to dosages using QCTOOL v2.0.2 (https://www.well.ox.ac.uk/∼gav/qctool_v2/), and the truth set genotypes to dosages using BCFtools v1.9 (Li, 2011). We then computed the correlation between the imputed dosages and the truth set dosages for all non-missing sites using a squared Pearson correlation coefficient (r^2^; squared output of the cor() function in R) across various AF bins. To determine the GT discordance rate between the imputed SNVs/INDELs and the truth set, we performed hard-calling with a genotype probability threshold of 0.90, setting all sites below this to missing using QCTOOL v2.0.2. Evaluation was restricted to sites shared across both the high-coverage and phase 3 panels. GT discordance is reported as the “1 minus precision” (obtained using RTG vcfeval (Cleary et al., 2015)) for each SGDP sample (n = 110). Precision was assessed using a mean of 3,178,927 ± 260,507 sites per sample from the phase 3 panel, and a mean of 3,231,687 ± 258,912 sites from the high-coverage panel. In the SNV/INDEL evaluation of the high-coverage panel stratified by variant type and genomic regions (Figure 5D), we assessed 45,124,785 SNVs and 2,185,638 INDELs in easy regions, and 13,352,708 SNVs and 3,806,176 INDELs in difficult regions. To compare SNV/INDEL imputation accuracy between the phase 3 and the high-coverage panels, we restricted evaluations to sites that are shared between the two panels, defined as sites with matching CHROM:POS:REF:ALT (40,088,294 SNVs and 2,249,447 INDELs; Figure 5E). SV imputation accuracy (r^2^) was assessed using the same approach described above for SNVs/INDELs (SV truth set described below). All imputed SVs were in HWE (exact test p value < 1e-10).

#### Generation of the SV truth set

To develop an SV truth set for use in imputation evaluations, SV-DELs and SV-INSs from the HGSVC freeze 4 (Ebert et al., 2021) were re-genotyped in the subset of 110 samples from the SGDP (Mallick et al., 2016) and sample NA24385 added as a control (sequenced internally using Illumina NovaSeq 6000) using both PanGenie v1.0.0 (Ebler et al., 2022) and Paragraph v2.4a (Chen et al., 2019). PanGenie was run with default parameters while Paragraph was run with one additional parameter (-M) set to 5 times the mean coverage of the sample. Genotypes from PanGenie were filtered for ‘high-gq’ (genotype quality ≥200) sites and then integrated with the PASS filter Paragraph genotypes into a single VCF with sites having discordant genotypes in the two callers converted to missing. To evaluate the quality of the integrated genotypes we applied the same approach to NA24385 and orthogonally validated the concordant genotypes (‘test set’) using the assembly based genotypes for NA24385 from the HGSVC (‘validation set’). The resulting genotype concordance, defined as the number of correct genotypes divided by the total number of sites in the validation (assembly) set, was 98.1% and the non-reference precision, defined as the number of correct heterozygous genotypes plus the number of correct homozygous alt genotypes divided by the total number of non-reference sites called in the test set, was 94.3%. The per-sample VCFs were then merged with BCFtools v1.15 (Danecek et al., 2021) into a multi-sample VCF, tagged with allele frequencies using bcftools and filtered to remove sites with missing alleles across all samples. The remaining SVs, having a valid genotype in at least one individual, were matched to SV-DELs and SV-INSs from the 1kGP high-coverage imputation panel. This was done to identify shared SV sites that could then be used to assess the accuracy of SV genotypes that were separately imputed using the high-coverage panel into the SGDP samples, as described in the section above. To identify corresponding SV events, both HGSVC and 1kGP VCF sites were converted to BED format by adding and subtracting a set window size (50 bp) from the SV start position. BED files were then compared using bedtools v2.26.0 (Quinlan and Hall, 2010) intersect with the “wao” parameter resulting in overlapping variants with a maximum distance of 100bp. Variant overlaps were then refined using a minimum length ratio of 80% (length of shorter SV/length of longer SV) to avoid matches between short and long SVs. Any sites that were monomorphic or deviating from HWE (exact test p value < 1e-10) in the multi-sample VCF were excluded, producing a final SV truth set of 7,573 sites (4,320 DELs and 3,253 INS).

#### External datasets

The following external datasets were used for evaluation purposes throughout the manuscript as described in the Results and STAR Methods sections above

**Table:** 

Dataset	Reference	Link	Sample information (downloaded file format)
GIAB v3.3.2	(Zook et al., 2019)	https://ftp-trace.ncbi.nlm.nih.gov/giab/ftp/release/NA12878_HG001/NISTv3.3.2/GRCh38/	Sample NA12878 (VCF and BED)
GIAB v4.2.1	(Wagner et al., 2022)	https://ftp-trace.ncbi.nlm.nih.gov/giab/ftp/release/NA12878_HG001/NISTv4.2.1/GRCh38/	Sample NA12878 (VCF and BED)
GIAB genome stratification region files v2.0	(Krusche et al., 2019)	https://ftp-trace.ncbi.nlm.nih.gov/giab/ftp/release/genome-stratifications/v2.0/GRCh38/union/	NA (BED)
Platinum Genome	(Eberle et al., 2017)	https://www.illumina.com/platinumgenomes.html	Sample NA12878 (VCF)
1kGP phase 3	(The 1000 Genomes Project Consortium, 2015)	http://ftp.1000genomes.ebi.ac.uk/vol1/ftp/release/20130502/	2,504 1kGP samples (VCF)
1kGP phase 3 GRCh38 SVs	(Sudmant et al., 2015)	http://ftp.1000genomes.ebi.ac.uk/vol1/ftp/phase3/integrated_sv_map/supporting/GRCh38_positions/	2,504 1kGP samples (VCF)
HGSVC	(Ebert et al., 2021)	http://ftp.1000genomes.ebi.ac.uk/vol1/ftp/data_collections/HGSVC2/release/	34 1kGP samples and sample NA24385 (VCF)
SGDP	(Mallick et al., 2016)	https://www.ebi.ac.uk/ena/browser/view/PRJEB9586	110 SGDP samples listed in Table S7 (FastQ)

### Quantification and statistical analysis

Details of exact analyses, statistical tests, and tools can be found in the main text and Methods.

## Consortia

The members of the Human Genome Structural Variation Consortium are Evan E. Eichler, Jan O. Korbel, Charles Lee, Tobias Marschall, Scott E. Devine, William T. Harvey, Weichen Zhou, Ryan E. Mills, Tobias Rausch, Sushant Kumar, Can Alkan, Fereydoun Hormozdiari, Zechen Chong, Yu Chen, Xiaofei Yang, Jiadong Lin, Mark B. Gerstein, Ye Kai, Qihui Zhu, Feyza Yilmaz, and Chunlin Xiao.

## Data Availability

• Data are shared via the International Genome Sample Resource (IGSR) (Fairley et al., 2020). Data can be accessed both through a website specific to this collection of data (https://www.internationalgenome.org/data-portal/data-collection/30x-grch38) and via displays integrating the data with other datasets generated both on the 1000 Genomes Project samples and additional openly consented samples. Within the IGSR data portal, files can be browsed by sample, sequencing technology, and population. Direct links to the IGSR FTP locations sharing FASTQs, CRAMs, GVCFs, SNV/INDEL VCFs, SV VCF, haplotype-resolved SNV/INDEL/SV VCFs, and a sample metadata file listing pedigree and sex information for the 3,202 sequenced samples can be found on the collection page. The data are also available at several other repositories including The European Bioinformatics Institute at the European Molecular Biology Laboratory (EMBL-EBI), the SNP database (dbSNP), and the database of Genomic Structural Variation (dbVar), where they can be browsed at the variant level, with accession numbers listed in the key resource table. The GRCh38 lifted-over version of the phase 3 1kGP SNV/INDEL call set, generated as part of this paper to facilitate comparative analysis, has been deposited at EMBL-EBI and IGSR FTP. • This paper does not report original code. • Any additional information required to reanalyze the data reported in this paper is available from the lead contact upon request.

## Supplemental information

Document S1. Tables S1–S6

Table S7. List of 110 SGDP samples used in the imputation performance evaluation, related to Figures 5 and 6

Document S2. Consortium affiliations

Data S1. Genomic DNA from 3,202 samples from the 1000 Genomes Project
