# Supplementary material for: High-coverage whole-genome sequencing of the expanded 1000 Genomes Project cohort including 602 trios
Source: Cell. 2022 Sep 1;185(18):3426–3440.e19. doi: 10.1016/j.cell.2022.08.004 (PMC9439720; doi:10.1016/j.cell.2022.08.004)
Supplement: Document S1. Tables S1–S6 [file mmc1.pdf]

Cell, Volume *185*

Supplemental information

High-coverage whole-genome sequencing of the expanded 1000 Genomes Project cohort including 602 trios

Marta Byrska-Bishop, Uday S. Evani, Xuefang Zhao, Anna O. Basile, Haley J. Abel, Allison A. Regier, André Corvelo, Wayne E. Clarke, Rajeeva Musunuri, Kshithija Nagulapalli, Susan Fairley, Alexi Runnels, Lara Winterkorn, Ernesto Lowy, Human Genome Structural Variation Consortium, Paul Flicek, Soren Germer, Harrison Brand, Ira M. Hall, Michael E. Talkowski, Giuseppe Narzisi, and Michael C. Zody

SUPPLEMENTAL TABLES

Table S1. Sample counts in the expanded (3,202-sample) and original (2,504-sample) 1kGP cohort stratified by population, sex, and pedigree status, related to Figure 1. Thirteen samples are part of 2 trios (hence only 1,793 unique samples contribute to the 602 trios; not 1,806), either because they are part of a multi-generational family, i.e. are a child in one trio and a parent in another trio (HG00702, NA19685, NA19675), and/or because they are a part of a quad (5 quads were included in total) that was broken down into 2 trios when pedigree-based correction was applied following haplotype phasing (HG00656, HG00657, HG03642, HG03679, HG03943, HG03944, NA19660, NA19661, NA19678, NA19679). Super-population ancestry groups: European (EUR), African (AFR), East Asian (EAS), South Asian (SAS), American (AMR). Populations: African Caribbean in Barbados (ACB), People with African Ancestry in Southwest USA (ASW), Esan in Nigeria (ESN), Gambian in Western Division, Mandinka (GWD), Luhya in Webuye, Kenya (LWK), Mende in Sierra Leone (MSL), Yoruba in Ibadan, Nigeria (YRI), Colombians in Medellin, Colombia (CLM), People with Mexican Ancestry in Los Angeles, CA, USA (MXL), Peruvians in Lima, Peru (PEL), Puerto Ricans in Puerto Rico (PUR), Chinese Dai in Xishuangbanna, China (CDX), Han Chinese in Beijing, China (CHB), Han Chinese South, China (CHS), Japanese in Tokyo, Japan (JPT), Kinh in Ho Chi Minh City, Vietnam (KHV), Utah residents (CEPH) with Northern and Western European ancestry (CEU), Finnish in Finland (FIN), British from England and Scotland, UK (GBR), Iberian Populations in Spain (IBS), Toscani in Italia (TSI), Bengali in Bangladesh (BEB), Gujarati Indians in Houston, TX, USA (GIH), Indian Telugu in the UK (ITU), Punjabi in Lahore, Pakistan (PJL), Sri Lankan Tamil in the UK (STU).

Population	Super-population	Sex (1=male, 2=female)	# across 3,202 samples	# across 2,504 samples	# in trios	No. of trios
ACB	AFR	1	57	47	30	20
		2	59	49	30	
ASW	AFR	1	33	26	20	13
		2	41	35	19	
ESN	AFR	1	84	53	71	43
		2	65	46	58	
GWD	AFR	1	93	55	91	58
		2	85	58	83	
LWK	AFR	1	44	44	0	0
		2	55	55	0	
MSL	AFR	1	50	42	16	11
		2	49	43	17	

YRI	AFR	1	97	52	92	56
		2	81	56	76	
CLM	AMR	1	58	43	48	35
		2	74	51	57	
MXL	AMR	1	43	32	40	32
		2	54	32	50	
PEL	AMR	1	54	41	47	35
		2	68	44	58	
PUR	AMR	1	70	54	51	35
		2	69	50	54	
CDX	EAS	1	44	44	0	0
		2	49	49	0	
CHB	EAS	1	46	46	0	0
		2	57	57	0	
CHS	EAS	1	86	52	80	51
		2	77	53	70	
JPT	EAS	1	56	56	0	0
		2	48	48	0	
KHV	EAS	1	60	46	34	21
		2	62	53	29	
CEU	EUR	1	87	49	84	57
		2	92	50	87	
FIN	EUR	1	38	38	0	0
		2	61	61	0	
GBR	EUR	1	46	46	0	0
		2	45	45	0	
IBS	EUR	1	81	54	77	50
		2	76	53	73	
TSI	EUR	1	53	53	0	0
		2	54	54	0	
BEB	SAS	1	60	42	41	30
		2	71	44	49	
GIH	SAS	1	56	56	0	0
		2	47	47	0	

ITU	SAS	1	61	59	4	3
		2	46	43	5	
PJL	SAS	1	77	48	65	42
		2	69	48	61	
STU	SAS	1	65	55	16	10
		2	49	47	10	
	**Total:**		**3,202**	**2,504**	**1,793**	**602**

Table S2. Mean SNV density per 1 kb of sequence in the 3,202-sample high coverage call set, related to Figure 1. Phased: SNV density in the phased high quality subset of SNV/INDEL calls; Genotyped: SNV density in the complete variant callset (based on VQSR PASS variants only).

Chromosome	SNV Density per 1kb region	
	Phased	Genotyped
chr1	21.88	36.46
chr2	23.89	40.16
chr3	23.89	40.11
chr4	24.39	41.3
chr5	23.76	39.91
chr6	23.99	39.88
chr7	24.61	41.1
chr8	25.5	42.84
chr9	21.76	36.66
chr10	24.77	41.15
chr11	24.11	40.61
chr12	23.65	39.82
chr13	24.22	41.04
chr14	23.93	40.06
chr15	23.52	39.21
chr16	24.78	41.43
chr17	23.39	39.02
chr18	23.25	39.6
chr19	26.62	43.21
chr20	24.23	40.34
chr21	22.84	37.77
chr22	25.03	41.41
chrX	17.04	30.16

Table S3. Average sample-level count of small variants per functional consequence category stratified by super-population, related to Figure 1. Super-population ancestry labels: European (EUR), African (AFR), East Asian (EAS), South Asian (SAS), American (AMR).

Filtering condition	Super-population	Functional consequence category				
		stop-gain	splice donor & acceptor	frameshift	missense	synonymous
No MAF filtering	All	76	314	195	10,625	11,956
	AFR	83	365	215	12,071	13,795
	EUR	73	287	188	9,974	11,154
	EAS	73	302	186	10,042	11,171
	SAS	74	296	190	10,232	11,454
	AMR	76	297	190	10,213	11,461
MAF ≤ 1%	All	11	18	14	754	569
	AFR	15	30	22	1,215	1,044
	EUR	9	12	10	540	353
	EAS	10	14	12	594	398
	SAS	10	14	12	648	453
	AMR	10	13	11	567	387

Table S4. Count of SV sites at the cohort and sample level, related to Figure 2. SV types: DEL: deletion, DUP: duplication, mCNV: multiallelic copy number variant, INS: insertion, INV: inversion, BND: breakends, CPX: complex SV, CTX: inter-chromosomal translocation.

SV TYPE	# SV sites across 3,202 samples			# SVs / sample		
	GATK-SV	svtools	Absinthe	GATK-SV	svtools	Absinthe
INS	48,333	75,283	7,183	3,019	1,761	2,270
DEL	89,445	65,184	-	3,783	3,417	-
DUP	26,353	10,594	-	990	459	-
INV	381	1,447	-	12	127	-
BND	82,218	26,152	-	-	2,188	-
CPX	3,624	-	-	216	-	-
CTX	16	-	-	1	-	-
mCNV	674	-	-	385	-	-
ALL	251,044	178,660	7,183	8,406	7,952	2,270

Table S5. Quality of SVs evaluated by PacBio support and inheritance, related to Figure 2. SV types: INS: insertion, DEL: deletion, DUP: duplication, INV: inversion.

	SV TYPE	All SVs		Call set specific SVs		SVs shared with other call sets		Integrated Call sets	
		**GATK-SV**	**Absinthe /svtools**	**GATK-SV**	**Absinthe /svtools**	**GATK-SV**	**Absinthe /svtools**	**High-cov**	**Phase3**
Proportion of VaPoR Supported SVs	INS	92.90%	97.60%	89.80%	96.30%	98.40%	99.00%	94.93%	93.94%
	DEL	88.00%	92.80%	71.40%	76.20%	92.60%	95.30%	90.07%	89.93%
	DUP	89.60%	88.10%	87.20%	62.70%	94.90%	95.40%	89.62%	22.22%
	INV	97.10%	47.60%	75.00%	44.80%	100%	55.70%	83.78%	53.90%
Overlap with PacBio call sets	INS	93.20%	97.70%	90.00%	96.60%	99.20%	99.00%	95.63%	97.55%
	DEL	90.50%	94.10%	72.10%	79.40%	96.90%	97.10%	95.85%	89.71%
	DUP	3.30%	4.50%	3.90%	8.10%	1.70%	2.50%	81.94%	2.14%
	INV	20.30%	18.10%	18.50%	13.70%	30.40%	32.50%	18.58%	24.63%
de novo Rate	INS	2.90%	1.90%	4.00%	1.70%	0.90%	2.10%	4.64%	NA
	DEL	4.70%	1.30%	10.60%	5.30%	2.60%	0.50%	2.09%	NA
	DUP	11.90%	0.50%	13.80%	1.30%	6.90%	0.00%	7.13%	NA
	INV	2.80%	11.30%	2.90%	13.70%	2.00%	3.60%	8.11%	NA

Table S6. Counts of variants passing specified filtering criteria, related to Figure 5 and 6. Variants passing all filtering criteria were included in haplotype phasing. PASS: sites with “PASS” in the FILTER column of the VCF, Miss.: genotype missingness; HWE: Hardy-Weinberg Equilibrium exact test p-value > 1e-10 in at least one of the five 1kGP super-populations; ME: mendelian error rate across complete trios; MAC: minor allele count. SNV: single nucleotide polymorphism, INDEL: short insertion or deletion, SV-DEL: large deletion, SV-INS: large insertion, SV-DUP: duplication, SV-INV: inversion.

Variant type	PASS	Miss.< 5%	HWE	ME ≤ 5%	MAC ≥ 2	All filters
SNV	110,905,285	109,770,155	118,635,957	117,788,297	72,632,423	64,109,737*
INDEL	14,421,124	16,642,357	18,849,519	18,452,006	14,074,540	9,516,672*
SV-DEL	90,259	88,981	89,237	88,662	57,396	54,074
SV-INS	49,693	47,493	48,832	49,183	35,672	32,548
SV-DUP	28,242	25,693	27,831	27,070	18,496	15,234
SV-INV	920	909	882	868	689	603

* 116,417 SNVs and 57,655 INDELs were excluded from the phased panel after haplotype phasing (see Methods). This post-phasing filtering resulted in the final count of 73,452,337 small variants (63,993,320 SNVs and 9,459,017 INDELs) and 102,459 SVs (none of the SVs had to be filtered out after phasing) included in the phased panel.
