# Supplementary material for: High-coverage whole-genome sequencing of the expanded 1000 Genomes Project cohort including 602 trios
Source: Cell. 2022 Sep 1;185(18):3426–3440.e19. doi: 10.1016/j.cell.2022.08.004 (PMC9439720; doi:10.1016/j.cell.2022.08.004)
Supplement: Document S2. Consortium affiliations [file mmc3.pdf]

Human Genome Structural Variation Consortium: Evan E. Eichler[1,2], Jan O. Korbel[3], Charles Lee[4], Tobias Marschall[5], Scott E. Devine[6], William T. Harvey[1], Weichen Zhou[7], Ryan E. Mills[7,8], Tobias Rausch[3], Sushant Kumar[9,10], Can Alkan[13,14], Fereydoun Hormozdiari[15], Zechen Chong[16], Yu Chen[16], Xiaofei Yang[4,17,18], Jiadong Lin[19], Mark B. Gerstein[9,10,11,12], Ye Kai[18,19], Qihui Zhu[4], Feyza Yilmaz[4], Chunlin Xiao[20].

1. Department of Genome Sciences, University of Washington School of Medicine, Seattle, WA 98195, USA.
2. Howard Hughes Medical Institute, University of Washington, Seattle, WA 98195, USA.
3. European Molecular Biology Laboratory, Genome Biology Unit, 69117 Heidelberg, Germany.
4. The Jackson Laboratory for Genomic Medicine, Farmington, CT 06032, USA.
5. Heinrich Heine University Düsseldorf, Medical Faculty, Institute for Medical Biometry and Bioinformatics, 40225 Düsseldorf, Germany.
6. Institute for Genome Sciences, University of Maryland School of Medicine, Baltimore, MD 21201, USA.
7. Department of Computational Medicine & Bioinformatics, University of Michigan, Ann Arbor, MI 48109, USA.
8. Department of Human Genetics, University of Michigan, Ann Arbor, MI 48109, USA.
9. Department of Molecular Biophysics and Biochemistry, Yale University, New Haven, CT 06520, USA.
10. Program in Computational Biology and Bioinformatics, Yale University, New Haven, CT 06520, USA.
11. Department of Computer Science, Yale University, New Haven, CT 06520, USA.
12. Department of Statistics & Data Science, Yale University, New Haven, CT 06520, USA.
13. Department of Computer Engineering, Bilkent University, Bilkent 06800 Ankara, Turkey.
14. Bilkent-Hacettepe Health Sciences and Technologies Program, Bilkent University, Bilkent 06800 Ankara, Turkey.
15. Department of Biochemistry and Molecular Medicine, MIND Institute and Genome Center, University of California Davis, Sacramento, CA 95817, USA.
16. Department of Genetics and Informatics Institute, Heersink School of Medicine, University of Alabama at Birmingham, Birmingham, AL 35294, USA.
17. School of Computer Science and Technology, Faculty of Electronic and Information Engineering, Xi’an Jiaotong University, Xi’an, Shaanxi 710049, China.
18. MOE Key Lab for Intelligent Networks & Networks Security, Faculty of Electronic and Information Engineering, Xi’an Jiaotong University, Xi’an, Shaanxi 710049, China.
19. School of Automation Science and Engineering, Faculty of Electronic and Information Engineering, Xi’an Jiaotong University, Xi’an, Shaanxi 710049, China.
20. National Center for Biotechnology Information, National Library of Medicine, National Institutes of Health, Bethesda, MD 20894, USA.
